# A systematic review of the effect of infrastructural interventions to promote cycling: strengthening causal inference from observational data

**DOI:** 10.1186/s12966-019-0850-1

**Published:** 2019-10-26

**Authors:** Famke J. M. Mölenberg, Jenna Panter, Alex Burdorf, Frank J. van Lenthe

**Affiliations:** 1 Department of Public Health, Erasmus MC, University Medical Center Rotterdam, P.O. Box 2040, 3000 CA Rotterdam, The Netherlands; 2 MRC Epidemiology Unit & UKCRC Centre for Diet and Activity Research (CEDAR), University of Cambridge, Cambridge, UK; 3 Department of Human Geography and Spatial Planning, Utrecht University, Utrecht, The Netherlands

**Keywords:** Cycling, Built environment, Natural experiments, Methodologies, Causal effects, Inequalities

## Abstract

**Background:**

Previous reviews have suggested that infrastructural interventions can be effective in promoting cycling. Given inherent methodological complexities in the evaluation of such changes, it is important to understand whether study results obtained depend on the study design and methods used, and to describe the implications of the methods used for causality. The aims of this systematic review were to summarize the effects obtained in studies that used a wide range of study designs to assess the effects of infrastructural interventions on cycling and physical activity, and whether the effects varied by study design, data collection methods, or statistical approaches.

**Methods:**

Six databases were searched for studies that evaluated infrastructural interventions to promote cycling in adult populations, such as the opening of cycling lanes, or the expansion of a city-wide cycling network. Controlled and uncontrolled studies that presented data before and after the intervention were included. No language or date restrictions were applied. Data was extracted for any outcome presented (e.g. bikes counted on the new infrastructure, making a bike trip, cycling frequency, cycling duration), and for any purpose of cycling (e.g. total cycling, recreational cycling, cycling for commuting). Data for physical activity outcomes and equity effects was extracted, and quality assessment was conducted following previous methodologies and the UK Medical Research Council guidance on natural experiments. The PROGRESS-Plus framework was used to describe the impact on subgroups of the population.

Studies were categorized by outcome, i.e. changes in cycling behavior, or usage of the cycling infrastructure. The relative change was calculated to derive a common outcome across various metrics and cycling purposes. The median relative change was presented to evaluate whether effects differed by methodological aspects.

**Results:**

The review included 31 studies and all were conducted within urban areas in high-income countries. Most of the evaluations found changes in favor of the intervention, showing that the number of cyclists using the facilities increased (median relative change compared to baseline: 62%; range: 4 to 438%), and to a lesser extent that cycling behavior increased (median relative change compared to baseline: 22%; range: − 21 to 262%). Studies that tested for statistical significance and studies that used subjective measurement methods (such as surveys and direct observations of cyclists) found larger changes than those that did not perform statistical tests, and those that used objective measurement methods (such as GPS and accelerometers, and automatic counting stations). Seven studies provided information on changes of physical activity behaviors, and findings were mixed. Three studies tested for equity effects following the opening of cycling infrastructure.

**Conclusions:**

Study findings of natural experiments evaluating infrastructural interventions to promote cycling depended on the methods used and the approach to analysis. Studies measuring cycling behavior were more likely to assess actual behavioral change that is most relevant for population health, as compared to studies that measured the use of cycling infrastructure. Triangulation of methods is warranted to overcome potential issues that one may encounter when evaluating environmental changes within the built environment.

**Trial registration:**

The protocol of this study was registered at PROSPERO (CRD42018091079).

## Background

Promoting physical activity is one of the key strategies to combat the burden of many chronic diseases [1]. Cycling can contribute to meeting the recommended daily physical activity levels [2, 3]. A meta-analysis including 187,000 individuals and 2.1 million person-years showed that 2.5 h per week of cycling at moderate intensity was associated with a 10% lower mortality risk, independent of overall levels of physical activity [4]. In addition to this, a Danish study found that those who cycled *and*, those who started cycling after the age of 50 years had a lower risk of coronary heart disease and developing diabetes than those who did not cycle [5, 6]. Modelling studies have also showed that the population health benefits of cycling outweigh the negative risks, such as exposure to air pollution and traffic accidents [7, 8]. This indicates that promoting cycling can result in population-level health benefits.

Providing an infrastructure that supports the needs of cyclists has been considered as an important strategy to encourage more cycling in cities [9–11]. However, designing studies to evaluate such infrastructural interventions is challenging. Although randomized controlled trials (RCTs) are regarded as the gold-standard for estimating causal effects of health interventions, to our knowledge no studies exist that used the RCT design to assess the impact of infrastructural interventions on cycling. This is not surprising, as changes in the built environment are often beyond control of the researcher and therefore difficult to randomize. Other analytical techniques are required to evaluate these so-called “natural experiments”, in which variation in accessibility to new cycling infrastructure is used to assign intervention and control groups [12–14].

Two recent systematic reviews have been completed which examine the impact of infrastructure on levels of cycling [15, 16]. Both reported that cycling increased following the introduction of new infrastructure, or upgrading of existing infrastructure. However, both reviews also noted that the methods in the included studies may have affected the study findings. Stappers and colleagues [15] noted variable quality in study designs across studies examining impacts on physical activity, active transport and sedentary behavior. They suggest that more refined designs may decrease the possibility of detecting intervention effects. Panter and colleagues [16] focused only on studies assessing walking and cycling, and examined the evidence for the effectiveness and mechanism of interventions. They found that higher quality studies were more likely to report intervention effects for cycling. Taken together, differences in methods may have impacted the overall conclusion (no changes vs positive changes), or the magnitude of the finding (small changes vs large changes). Ignoring methodological differences may wrongly lead to the conclusion that some interventions were more effective than others.

The current review builds on the main finding of previous reviews that interventions in the built environment may affect cycling [15, 16]. We focused on the methodological approaches undertaken to evaluate the effects of infrastructural interventions. Both reviews did not quantitatively summarize the findings, thereby leaving the question unanswered if the magnitude of the findings changed when using different methodology. One review was unable to capture relevant literature published outside of health-related journals [15]. The research questions are likely to be different between health researchers and transportation researchers, potentially leading to differences in study designs and findings.

Focusing on whether different methodological approaches produce different results, and assessing the strengths and limitations of different methods for causality, will provide greater understanding about the implications of findings from research and their utility for policy makers and practitioners. Therefore, the aims of this systematic review were to summarize the effects of infrastructural interventions on cycling and physical activity in the population, and to evaluate whether the effects varied by study design, data collection methods, or statistical approaches.

## Methods

The protocol of this study was registered in March 2018 at PROSPERO (CRD42018091079). Our systematic literature search followed the Preferred Reporting Items for Systematic Reviews and Meta-Analyses (PRISMA) guidelines [17].

### Search strategy

Various electronic databases (Embase.com, Medline Ovid, Web of Science, PsycINFO Ovid, CINAHL EBSCOhost, Google scholar) were searched for literature published until February 2018 for any studies assessing infrastructural projects to promote cycling. We updated the initial search until June 2019 to additionally include most recent publications. Search terms for the different databases can be found in Additional file 1. Search terms were constructed of 3 parts, including synonyms for cycling infrastructure to identify exposures, synonyms for cycling behavior, active transport, physical activity and lifestyle changes to identify outcomes, and a term that excluded conference abstracts, letter to the editors, notes and editorials. No restrictions were made on language. Database searches were supplemented with searches of reference lists of included studies and key review papers.

### Study selection and inclusion criteria

All titles and abstracts identified during the initial search were screened for inclusion by two independent researchers (FJMM, NB). Additional articles identified through the updated search were screened by a single author (FJMM). After screening titles and abstracts, full-text articles were screened according to predefined criteria. Articles obtained in full-text were reassessed for inclusion by the first two authors (FJMM, JP), and discrepancies were resolved after discussion with a third researcher (FJvL). Eligibility criteria included: 1) a study evaluating an infrastructural intervention to promote cycling, 2) any measure of cycling as outcome, 3) cycling measured before and after the intervention, and 4) reporting on a general adult population aged 16 years and above. Examples of interventions include the opening of cycling lanes, the installation of a city-wide cycling network, and the improvement of existing cycling infrastructure. We included papers that evaluated the same intervention, but reported on different outcomes or used different datasets or methods to collect outcome data. Controlled and uncontrolled studies were included to allow for a large variety of study designs. Studies were classified as controlled studies if data was collected in a different population that was selected based on comparable individual or neighborhood characteristics, and if similar data collection methods were used. We also classified studies as controlled studies if a comparison was made within the study population between people who lived closer to an intervention and those who lived further away. Studies that presented city- or area-wide cycling trends as a comparison were considered uncontrolled, as the data collection methods used in routine monitoring surveys often differed from that used in the intervention group, and population characteristics often differed between areas.

Studies that evaluated the introduction of cycling infrastructure together with other environmental components were included (i.e. bike parking, showers, rental bikes), as long as the main goal of the intervention was to promote cycling. Environmental interventions that did not change the cycling infrastructure were excluded. We specifically aimed to study population-based approaches to change health behaviors, and therefore excluded infrastructural interventions that were part of a combined intervention with behavioral components targeting the behavior of individuals (i.e. cycling courses, safety lessons, or other approaches that target individual behaviors). Studies that included media campaigns along the intervention were included, as long as they aimed to target the population as a whole.

We excluded opinion articles, qualitative evaluations without quantitative assessment, studies retrospectively collecting data on cycling, and studies not directly linked to an infrastructural intervention. We also excluded studies in which the presented outcome measure was not specified for cycling, like active travel which combined walking and cycling together, or modal shifts where the shift in mode was not specified.

### Data extraction

From the included studies, one researcher extracted data (FJMM) using a standardized data extraction form, and a second reviewer (JP) verified a 20% sample of the extracted data. The extracted data included publication details, description of the intervention, study design, data collection methods, analytical methodology, and study results.

Ideally, we would have extracted a single outcome related to cycling per study. However, most studies did not specify a primary outcome of cycling. Therefore, we extracted all cycling outcomes presented from the maximally adjusted model with the longest exposure time. We extracted all outcomes for various purposes of cycling (e.g. total cycling, recreational cycling, cycling for commuting), and all outcomes for various metrics of cycling (e.g. bike count data, cycling frequency, cycling duration). If the outcome was assessed in multiple populations or at multiple locations, we extracted the average change in cycling that was presented by the authors. If no summary measure was presented, we calculated an unweighted average effect. Some studies stratified the population by exposure status, and evaluated a possible exposure-outcome relationship by distance from home to the intervention or usage of the intervention. All available information was extracted for these studies and included in the descriptive part of the review. However, including all strata-specific outcomes in the quantitative analyses would mean that studies with multiple strata would have a much greater contribution to the findings than studies without stratification. Therefore, we only used the results from the group most likely to use the intervention in the quantitative summary (e.g. smallest distance or largest potential usage). We noted that various metrics were used for expressing data relevant to cycling. We distinguished outcomes that evaluated cycling behavior (e.g. making a bike trip, cycling frequency, cycling duration) from those that evaluated usage of cycling infrastructure (e.g. bikes counted in the city, bikes counted on the new infrastructure). We extracted data on both absolute change (no fixed unit, can refer to various metrics) and relative change (expressed as percentage change over time) in cycling between before and after measurements, and attempted to calculate outcomes for both where possible. We used a similar framework presented by Goodman [18] to compute measures of absolute and relative change. Outcomes expressed as ratios were interpreted as relative changes. For uncontrolled studies, the relative change was computed by dividing the absolute change by the baseline level of cycling in the study sample. For controlled studies, we first computed the relative change in the intervention and control group separately. Subsequently, the calculated relative change in the intervention group was divided by the calculated relative change in the control group. Likewise, to obtain an absolute change when only relative changes were presented, we multiplied the relative change by the baseline estimate in the study sample as a whole for uncontrolled studies, and by the baseline estimate in the control group for controlled studies. Examples of the data extracted and how outcomes were calculated are presented in Additional file 2. Authors were contacted if only the direction of the association was presented. For each study we extracted data on statistical tests performed, and if significant results were found (*P* < 0.05). However, we focused on directions of the association rather than significance, since a substantive part of the studies did not test for significant changes in cycling outcomes that were of interest for this review.

We extracted data on the methodological quality, and on all design elements and additional analyses that may have supported causal inference following previous methodologies. The quality items described by Ogilvie et al. [19] were extracted, which used the criteria from the Community Guide of the US Task Force on Community Preventive Services to assess study design [20], and criteria developed for the Effective Public Health Practice Project in Hamilton, Ontario to score five items related to the quality of the research performed [21]. The five items included representativeness, comparability, credibility of data collection instruments, retention, and attributability of the effect to the intervention. The original instrument also assessed randomization, but this was not assessed as the allocation to the intervention and comparison group was not under control of the researcher. In addition, we extracted the results from additional analyses that may support causal inference identified by the UK Medical Research Council guidance on natural experiments [12], including multiple comparison groups, the inclusion of a neutral outcome that is not expected to change as a consequence of the new cycling infrastructure, and the use of complementing research methodologies.

The PROGRESS-Plus framework was used to describe the impact of the infrastructural interventions on subgroups of the population [22]. The PROGRESS-Plus framework considers nine factors for which differences in effect may occur: 1) place of residence, 2) race, ethnicity, culture, language, 3) occupation, 4) gender, sex, 5) religion, 6) education, 7) socioeconomic status, 8) social capital, and 9) the ‘Plus’-factor that could be other characteristics associated with social disadvantage. In our study we considered age, health status or BMI, bike ownership, and car ownership as Plus-factors, since these factors may have been relevant determinants of disadvantage given the context of the intervention.

### Data synthesis

We provided a descriptive narrative synthesis of studies. There was no possibility to quantitatively summarize the results, because of the large variety of outcome metrics and purposes of cycling presented, the lack of a primary outcome, and the lack of a common outcome across studies. Therefore, we presented the median relative change for the umbrella-terms*cycling behavior* and *infrastructure usage* for all studies, and by study design (controlled vs uncontrolled; exposure time ≥ 1 year vs < 1 year), data collection methods (objective vs subjective), and analytical approaches (tested vs not tested)*.* We did not present units for the median relative change because it can refer to various metrics. For example, an increase in cycling behavior of 30% could refer to an increase in the proportion of cyclists, cycling frequency, or cycling duration.

An overview of studies with baseline characteristics or performed adjusted analyses by any of the PROGRESS-Plus factors was presented. We provided a descriptive narrative synthesis for the studies that formally tested for differential effects on PROGRESS-Plus factors.

## Results

### Study characteristics

**Fig. 1:**
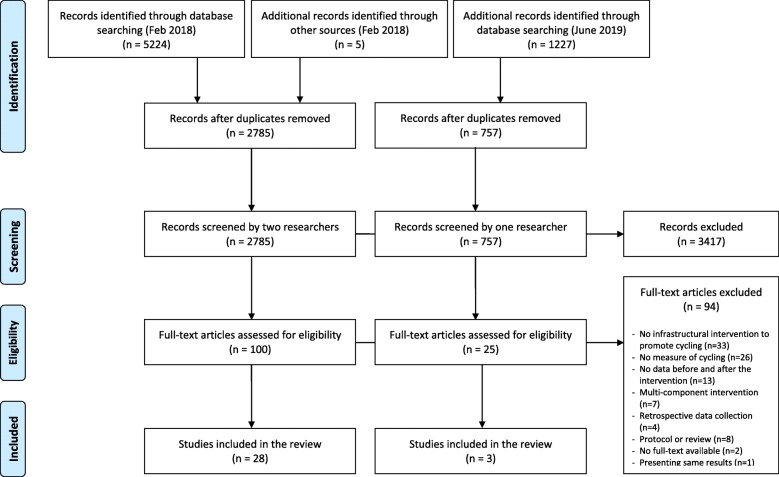
Flow diagram of study selection

From the 3542 potential records, 125 full-text articles were screened and this resulted in 31 studies (29 interventions) from 11 countries that met the eligibility criteria (Fig. 1). The major reason for exclusion of full-text articles is presented in Additional file 3. Table 1 presents the characteristics of included studies categorized by the outcome of interest. Twenty studies presented data on cycling behavior [23–42], and 16 studies assessed usage of the cycling infrastructure [23, 29, 31, 38, 42–53]. All infrastructural interventions were conducted in urban areas in high-income countries. The interventions were very diverse in terms of design and scale, ranging from the introduction of a cycling bridge, single or multiple cycle paths or lanes, or a city-wide cycling network. Six studies (5 interventions) described issues related to data collection due to delays in the construction work, resulting in shorter follow-up periods than planned [23, 31–34, 39]. In addition to this, three studies (2 interventions) mentioned that the intervention was not fully completed within the study time frame [31, 33, 34]. Most studies used a similar analytical approach by comparing a single estimate before the intervention with a single estimate after the intervention, with or without comparing it to changes in a control group. One study used a fixed-effects approach to evaluate the within-person change over time [27], and three studies tested if there was a significant interaction between the intervention and time [29, 31, 35]. One study conducted an interrupted time series analyses, whereby the date of the opening of the cycling track was used to set the time of interruption [47].

**Table 1:** Study characteristics of infrastructural interventions to promote cycling

Reference (country)	Infrastructural intervention	Controlled comparison	Type of comparison	Data collection method; time between measurements; time exposed	Outcome studied	Analytical methodology	Confounders	Direction of the results; significance; absolute (A) and relative (R) change
Cycling behavior								
Aittasalo [23] (Finland)	Environmental improvements made to the main and connecting walking and cycling paths	No	Employees working at workplaces in the area where new infrastructure was introduced	Survey Time between measurements; 18–24 months Time exposed; 2 months	Cycling frequency as part of the journey to work (days/week)	Difference over time, tested by Wilcoxon Signed Rank Test	Unadjusted	Not in favor of the intervention, not significant A: Not applicable R: Not applicable
					Cycling distance as part of the journey to work (km/trip)			Not in favor of the intervention, not significant A: Not applicable R: Not applicable
					Cycling time as part of the journey to work (min/trip)			Not in favor of the intervention, not significant A: Not applicable R: Not applicable
					Cycling frequency as part of the journey from work (days/week)			Not in favor of the intervention, not significant A: Not applicable R: Not applicable
					Cycling distance as part of the journey from work (km/trip)			Not in favor of the intervention, not significant A: Not applicable R: Not applicable
					Cycling time as part of the journey from work (min/trip)			Not in favor of the intervention, not significant A: Not applicable R: Not applicable
Aldred [24] (UK)	Infrastructural interventions in 3 neighborhoods, transforming local environments for walking and cycling	Yes	Residents living in the intervention areas vs control areas	Travel diary Time between measurements; 12 months Time exposed; not specified but could range from 1 to 12 months	Made a bike trip in the past week (yes-no)	Difference-in-difference, tested by regression models	Demographic variables, socioeconomic variables, health indicator, and car ownership	In favor of the intervention, not significant A: 3.2%-point R: 16%
					Cycling time (min/week)			In favor of the intervention, not significant A: 4 min/week R: 14%
		Yes	Residents living in low-dose or high-dose areas (defined by stakeholders involved in implementation) in the intervention areas vs control areas	Travel diary Time between measurements; 12 months Time exposed; not specified but could range from 1 to 12 months	Made a bike trip in the past week (yes-no)	Difference-in-difference, tested by regression models	Demographic variables, socioeconomic variables, health indicator, and car ownership	All comparisons in favor of the intervention Low-dose area: not significant A: 0.7%-point R: 10% High-dose area: significant A: 7.2%-point R: 24%
					Cycling time (min/week)			All comparisons in favor of the intervention: Low-dose area: not significant A: 1 min/week R: 5% High-dose area: not significant A: 9 min/week R: 30%
Brown [25] (US)	Complete street intervention including the completion of an incomplete bike lane (10.7 km), connecting the airport to down town districts	Yes	Residents living near (≤0.8 km) vs far (0.8–2 km) from the new infrastructure	GPS and accelerometers Time between measurements; 12 months Time exposed; 1–8 months	Made a bike trip on the intervention road (yes-no)	Difference-in-difference, but no statistical test conducted	Demographic and socioeconomic variables	Not in favor of the intervention, significance not tested A: 0%-point R: −11%
Brown [26] (US)	Same as above	No	Residents living within 2 km of the new infrastructure	GPS and accelerometers Time between measurements; 12 months Time exposed; 1–8 months	Cycling time on the intervention road among those who cycled (min/week)	Difference tested by paired t-test	Unadjusted	In favor of the intervention, not significant A: 7 min/week R: 38%
					Cycling time off the intervention road among those who cycled (min/week)			In favor of the intervention, not significant A: 6 min/week R: 15%
Burbidge and Goulias [27] (US)	Installation of a multi-use trail, creating a 4-km loop connecting two currently existing sidewalks, serving as transportation and recreation facility	No	Residents living within 1.6 km of the new infrastructure	Travel diary Time between measurements; 12 months Time exposed; 5 months	Total cycling trips (trips/day)	Difference tested by fixed effects regression models	Not reported	In favor of the intervention, not significant A: 0.01 trips/day R: 33%
Chowdhury [28] (New Zealand)	Introduction of a 3 cycle ways linking suburbs with the central business district, and the associated promotional campaigns	No	Residents living in the city where the new infrastructure was introduced	Survey Time between measurements; 4 years Time exposed; 12 months	Cycling at least weekly (yes-no)	Difference, but no statistical test conducted	Unadjusted	In favor of the intervention, significance not tested A: 10%-point R: 40%
Crane [29] (Australia)	A new cycle way (2.4 km) linking a new urban renewal area with the central business district	Yes	Residents living in the intervention area (suburbs surrounding the cycle way) vs a control area (matched for demographic characteristics)	Survey Time between measurements; 23–25 months Time exposed; 15–17 months	Cycling at least weekly (yes-no)	Difference-in-difference tested by regression models that included a two-way interaction term between time and proximity	Demographic variables	In favor of the intervention, not significant A: 44%-point R: 179%
		Yes	Residents living closer (< 1 km, 1–3 km) vs further (> 3 km) from the new infrastructure	Travel diary Time between measurements; 23–25 months Time exposed; 15–17 months	Cycling duration (min/week)			Those living < 1 km of the intervention: not in favor of the intervention, not significant A: −37 min/week R: −21% Those living 1–3 km from the intervention: in favor of the intervention, significant A: 96 min/week R: 54%
Deegan [30] (UK)	Extension of a city-wide cycling network aiming for 900 km, unfinished	No	Residents living in 31 intervention areas. Area-wide cycling trends in 2 control areas are presented for comparison	Survey (census data) Time between measurements; 10 years Time exposed; not specified but could range from 1 to 10 years	Proportion of commuting trips made by bike (%)	Difference-in-difference, but no statistical test conducted	Not reported	In favor of the intervention, significance not tested Average in 31 areas: A: not reported R: 87% Average in 2 control areas: A: not reported R: 75%
Dill [31] (US)	Installation of 8 bicycle boulevards (1.4 km to 6.7 km long)	Yes	Residents living within 0.3 km of the 8 intervention streets vs residents living within 0.3 km of the 11 control streets (selected to be similar in urban form and demographic characteristics)	GPS and accelerometers Time between measurements; 12 months Time exposed; 2–12 months	Cycling at least 10 min a day (yes-no)	Difference-in-difference tested by regression models that included a two-way interaction term between treatment and period	Demographic variables, weather conditions, distance to downtown, bike attitudes and car safety attitudes	In favor of the intervention, not significant A: 9%-point R: 22%
					Cycling time (min/day) for those cycling at least 10 min/day			Not in favor of the intervention, significant A: − 1 min/day R: − 1%
					Made a bike trip (yes-no)			Not in favor of the intervention, not significant A: −8%-point R: −15%
					Number of bike trips (trips/day) for those that made a bike trip			Not in favor of the intervention, not significant A: −0.4 trips/day R: −9%
Evenson [32] (US)	Extension of an existing trail (4.5 km), along with a spur (3.2 km) passing by schools, shopping areas, apartment buildings, and residential areas	No	Residents living in census blocks that are crossed by the intervention	Telephone interview Time between measurements; 19–28 months Time exposed; 2 months	Median cycling time (min/week)	Difference tested by Wilcoxon nonparametric test for differences	Unadjusted	Not in favor of the intervention, not significant A: 0 min/week R: 0%
					Median cycling time for transportation (min/month)			Not in favor of the intervention, not significant A: 0 min/week R: 0%
Goodman [33] (UK)	Construction of new walking and cycling infrastructure and improvement of existing routes in 3 cities plus a modest amount of promotion activities	Yes	Residents living within 5 km of the new infrastructure using proximity for comparison (per 1 km closer to the intervention)	7-day recall instrument Time between measurements; 24 months Time exposed; 7–21 months	Cycling time for transport (min/week)	Difference-in-difference tested by regression models	Demographic variables, socioeconomic variables, health indicator, and car ownership	Not in favor of the intervention, not significant A: −0.2 min/week R: not reported
				Survey	Cycling time for recreation (min/week)			In favor of the intervention, significant A: 2.5 min/week R: not reported
Song [34] (UK)	Same as above	No	Residents living within 5 km of the new infrastructure	7-day recall instrument Time between measurements; 24 months Time exposed; 7–21 months	Cycling time for utility purpose (min/week)	Difference over time tested by paired sample t-test	Unadjusted	In favor of the intervention, not significant A: 0.4 min/week R: 2%
					Cycling distance for utility purpose (km/week)			In favor of the intervention, not significant A: 0.4 km/week R: 7%
Hirsch [35] (US)	Expansion of two trails (16.3 km), including a bicycle and pedestrian bridge connecting residential areas to employment centers downtown and at the university	No	Residents living in 116 areas of the city with the new infrastructure. Historical time trends are presented for comparison	Survey (census data) Time between measurements; 10 years Time exposed; not specified but could range from 3 to 10 years	Proportion of workers who commuted by bike (%)	Difference over time, but no statistical test conducted	Not reported	In favor of the intervention, significance not tested A: 2.3%-point R: 130% Historical trend: A: 0.1%-point R: not reported
		Yes	Residents living in 116 areas of the city with the new infrastructure using distance to the intervention for comparison (results presented for the 25th, 50th and 75th percentiles)		Proportion of workers who commuted by bike (%)	Difference-in-difference tested by regression models that included a two-way interaction term between time and treatment	Demographic variables, socioeconomic variables, cycling infrastructure characteristics, total work-related trips, proportion of trips that cross the trail system	All comparisons in favor of the intervention, and all significant 25th percentile (1.1 km): A: 2.0%-point R: 115% 50th percentile (2.8 km): A: 1.9%-point R: 107% 75th percentile (5.9 km): A: 1.6%-point R: 92%
		Yes	Residents living in 116 areas of the city with the new infrastructure using proportion of commuting trips crossing the trail for comparison (results presented for the 25th, 50th and 75th percentiles)		Proportion of workers who commuted by bike (%)	Difference-in-difference tested by regression models that included a two-way interaction term between time and treatment	Demographic variables, socioeconomic variables, cycling infrastructure characteristics, total work-related trips, distance to the trail	All comparisons in favor of the intervention, and all significant 25th percentile (11%): A: 1.0%-point R: 54% 50th percentile (29%): A: 1.9%-point R: 107% 75th percentile (42%): A: 2.6%-point R: 146%
		Yes	Residents living in 116 areas of the city with the new infrastructure using the joined effect of distance and trips crossing the trail for comparison		Proportion of workers who commuted by bike (%)	Difference-in-difference tested by regression models that included a two-way interaction term between time and treatment	Demographic variables, socioeconomic variables, cycling infrastructure characteristics, total work-related trips, proportion of trips that cross the trail system, distance to the trail	In favor of the intervention The increase in bicycle commuting was restricted to tracts that were close to the intervention, and had a higher proportion of commuting trips that crossed the trails
Krizek [36] (US)	Installation of multiple bicycle facilities and major bridge improvements to enhance accessibility to major employment centers	No	Residents living in areas within 1.6 km of the geographical centroids of a new facility. Area-wide cycling trends are presented for comparison	Survey (census data) Time between measurements; 10 years Time exposed; not specified but could range from 1 to 10 years	Bicycle mode share (%)	Difference tested by regression models	Not reported	In favor of the intervention, significant A: 0.2%-point R: 14% Whole area: A: 0.02%-point R: 5%
			Residents living in areas within 1.6 km of the geographical centroids of a new facility, or within 0.8 km from the endpoints of a facility					In favor of the intervention, significant A: 0.5%-point R: 46%
		No	Bicycle mode share crossing the river. Cycling trends that remained on the same side of the river are presented for comparison	Survey (census data) Time between measurements; 10 years Time exposed; not specified but could range from 1 to 10 years	Bicycle mode share crossing the river (%)	Difference tested by regression models	Not reported	In favor of the intervention, significant Crossing river: A: 1.6%-point R: 52% Average that remained at the same side of the river: A: 0.6%-point R: 28%
Lanzendorf [37] (Germany)	Cycling infrastructure improvements and marketing campaigns in 4 cities	No	Residents living in cities with the new infrastructure. Cycling trends in big cities are presented for comparison	Survey enriched with regional data Time between measurements; 6 years Time exposed; not specified but could range from 1 to 6 years	Cycling frequency (trips/day)	Difference over time, tested by Mann-Whitney U-test	Not reported	In favor of the intervention, significant Average of 4 cities: A: 0.07 trips/day R: 27% Big cities: A: 0.09 trips/day R: 31%
					Bicycle mode share (%)	Difference over time, but no statistical test conducted	Not reported	In favor of the intervention, significance not tested Average of 4 cities: A: 1.8%-point R: 21% Big cities: A: 2.4%-point R: 24%
Merom [38] (US)	The construction of a cycle way (16.5 km) and the associated promotional campaigns	Yes	Residents living near (< 1.5 km) vs far (1.5–5 km) from the new infrastructure	Telephone interviews Time between measurements; 4 months Time exposed; 3 months	Cycling time among those who cycled (min/week)	Difference-in-difference tested by ANOVA	Unadjusted	In favor of the intervention, significant A: 26 min/week R: 147%
Panter [39] (UK)	New bus network and an adjacent traffic-free walking and cycling route (22 km)	Yes	Residents working in the city with the new infrastructure, and living within ~ 30 km of work using proximity for comparison (results presented comparing those living 4 km from the intervention vs 9 km)	7-day recall instrument Time between measurements; 3 years Time exposed; 9–14 months	Likelihood of an increase in cycling time for commuting (yes-no)	Difference-in-difference tested by regression models	Demographic variables, socioeconomic variables, health indicators, car ownership and work related variables	In favor of the intervention, significant A: 87 min/week (among those who reported more cycling for commuting at follow-up) R: 34%
				Survey	Likelihood of an increase in total cycling time (yes-no)			In favor of the intervention, significant A: 115 min/week (among those who reported more cycling at follow-up) R: 32%
Pedroso [40] (US)	Infrastructure expansion in bicycle lanes (147 km) and improvements in bicycle signage, parking, and cyclist awareness, and the addition of a bike share program	No	Residents living in the city with the new infrastructure	Survey (census data) Time between measurements; 9 years Time exposed; not specified but could range from 1 to 7 years	Proportion of workers who commuted by bike (%)	Difference over time tested by regression models	Not reported	In favor of the intervention, significant A: 1.5%-point R: 167%
Smith [41] (US)	Bicycle lane expansion (> 160 km), and the introduction of bicycle share programs	No	Residents living in the city with the new infrastructure	Survey (census data) Time between measurements; 5 years Time exposed; 4 years	Number of cyclist	Difference over time tested by t-test	Not reported	In favor of the intervention, significant A: 4388 cyclist R: 262%
Wilmink and Hartman [42] (The Netherlands)	Improvements to an existing cycle route network, creating a comprehensive and interconnected network	No	Residents living in two neighborhoods with the new infrastructure	Home interview Time between measurements; 3 years Time exposed; not specified but could range from 1 to 3 years	Proportion of trips made by bike (%)	Difference-in-difference, no statistical test conducted	Not reported	In favor of the intervention, significance not tested A: 3%-point R: 7%
		Yes	Residents living in two neighborhoods with the new infrastructure vs one control neighborhood without the new infrastructure		Cycling frequency (trips per person per day)			In favor of the intervention, significance not tested A: not reported R: 4%
					Cycling distance (distance per person per day)			In favor of the intervention, significance not tested A: not reported R: 8%
Usage of the infrastructure								
Aittasalo [23] (Finland)	Environmental improvements made to the main and connecting walking and cycling paths	No	4 locations in the study area	Automatic counters Time between measurements; 24 months Time exposed; 2 months	Bikes per day during afternoon peak hour	Difference, no statistical test conducted	Not reported	In favor of the intervention, significance not tested Average of the 4 locations: A: 367 bikes/peak hour R: 57%
Barnes [43] (US)	Complete street redesign of a gateway to the university to improve the conditions for non-motorized users	No	1 location on the study road, for 2 directions of travel	Direct observation Time between measurements; 6 months Time exposed; not specified but could range from 1 to 6 months	Bikes per hour	Difference, no statistical test conducted	Not reported	In favor of the intervention, significance not tested Average of the 2 directions: A: 63 bikes/hour R: 83%
Crane [29] (Australia)	A new cycle way (2.4 km) linking a new urban renewal area with the central business district	No	2 locations on the study road. City-wide cycling trends and historic time trends are presented for comparison	Automatic counters Time between measurements; 36 months Time exposed; 16 months	Bikes per day during peak hours (6 h/day)	Difference, no statistical test conducted	If adjusted, estimated were adjusted for population growth	In favor of the intervention, significance not tested Average of the 2 locations: A: 144 bikes/peak hours (unadjusted) R: 4% (adjusted) City as a whole: A: −80 bikes/peak hours (unadjusted) R: −2% (adjusted) Historical trend: A: 300 bikes/peak hours (unadjusted) R: 126% (unadjusted) Historical trend, city as a whole: A: 300 bikes/peak hours (unadjusted) R: 111% (unadjusted)
Dill [31] (US)	Installation of 8 bicycle boulevards (1.4 km to 6.7 km long)	No	10 locations on the study roads	Method not described Time between measurements; 3 years Time exposed; 18 months	Number of bikes	Difference, but no statistical test conducted	Not reported	In favor of the intervention, significance not tested Average of the 10 locations: A: not reported R: 22%
Fitzhugh [44] (US)	Retrofıtting a neighborhood with an urban trail (4.6 km) that enhanced connectivity to retail and school destinations	Yes	1 location in the intervention neighborhood vs 2 locations in 2 control neighborhoods (matched along socioeconomic dimensions)	Direct observation Time between measurements; 2 years Time exposed; 14 months	Median number of bikes per 2 h	Difference-in-difference tested by Wilcoxon rank sums test	Not reported	In favor of the intervention, significant A: 2.2 bikes/2 h R: 224%
Goodno [45] (US)	The installation of two linked bicycle facilities serving downtown	No	4 locations on the study roads. City-wide cycling trends are presented for comparison	Methods not described; Time between measurements; 18–20 months Time exposed; 7–12 months	Bikes during peak hour	Difference, but no statistical test conducted	Not reported	In favor of the intervention, significance not tested Average of 4 locations: A: 124 bikes/peak hour R: 438% City as a whole: A: 20 bikes/peak hour R: 32%
Hans [46] (Denmark)	Improvements made to two large, interconnected bicycle infrastructures (18 km and 15 km) in city suburbs to enhance connectivity	No	2 locations on the study roads	Automatic counters, calibrated by visual counts Time between measurements; 35 months Time exposed; 16–22 months	Bikes per hour on weekdays during the rush hour in day light	Difference over time, but no statistical test conducted	Seasonal, weather and temporal variables	In favor of the intervention, significance not tested Average of the 2 locations: A: 43 bikes/hour R: 47%
					Bikes per hour on weekdays during the rush hour in dark			In favor of the intervention, significance not tested Average of the 2 locations: A: 38 bikes/hour R: 72%
					Bikes per hour on weekdays during the non-rush hour in day light			In favor of the intervention, significant Average of the 2 locations: A: 11 bikes/hour R: 19%
					Bikes per hour on weekend days in day light			In favor of the intervention, significant Average of the 2 locations: A: 10 bikes/hour R: 29%
Heesch [47] (Australia)	The opening of three new segments of a cycling lane (1.4 km, 0.9 km, 2.3 km) connecting the suburbs and the city center	No	1 location on the study road before the intervention, 2 locations on the study road after the intervention	Direct observation Time between measurements; 4 years and 1 month Time exposed; 3–38 months	Bikes per 2.5 h	Difference over time, but no statistical test conducted	Not reported	In favor of the intervention, significance not tested A: 376 bikes/2.5 h R: 276%
	The opening of the last segment of a cycling lane (2.3 km) connecting the suburbs and the city center	Yes	GPS tracking information on the study road vs 3 other routes surrounding the intervention	Mobile phone application Time between measurements; 1 year Time exposed; 6 months	Trend in monthly bike trips on the intervention road	Interrupted time-series	Seasonal variables	In favor of the intervention, significant A: 225 bike trips/month R: not applicable
		No	GPS tracking information on the major routes between suburbs and city center, including the intervention		Trend in monthly bike trips between suburbs and the city center			In favor of the intervention, significant A: 90 bike trips/month R: 102%
Law [48] (UK)	The introduction of superhighways for cyclists creating continuous cycling routes in the city center, and a public bike sharing system	No	21 locations in the intervention area	Direct observations (before) and automatic counters (after) Time between measurements; 9 years Time exposed; not specified but could range from 1 to 9 years	Bikes per hour	Difference over time, test not described	Not reported	In favor of the intervention, significant Average of the 21 locations: A: 154 bikes/hour R: 432%
Marques [49] (Spain)	Introduction of a cycling network in the city (164 km)	No	2000–2005: data from 2006 extrapolated 2006–2010: counts made in the city 2011–2013: algorithm based on count data and the number of rental bikes	Count data, changing methodology over time Time between measurements; 14 years Time exposed; not specified but could range from 1 to 7 years	Million bike trips per year	Difference, no statistical test conducted	Seasonal variables	In favor of the intervention, significance not tested A: 13.3 million trips/year R: 435%
McCartney [50] (UK)	Construction of a new pedestrian and cyclist bridge across the river towards the city center	No	5 locations to enter the city from the side of the bridge. City-wide cycling trends are presented for comparison	Direct observation; Time between measurements; 4 years Time exposed; 2 years	Bikes counted per 2 days	Difference over time, but no statistical test conducted	Not reported	In favor of the intervention, significance not tested Average of the 5 locations: A: 500 bikes/2 days R: 62% Rest of the city: A: 1700 bikes/2 days R: 48%
Merom [38] (US)	The construction of a cycle way (16.5 km) and the associated promotional campaigns	No	4 locations along the new infrastructure	Automatic counters Time between measurements; 5 months Time exposed; 3 months	Bikes per day	Difference, tested by regression models	Weather variables, day of the week and holiday season	In favor of the intervention, significant Average of the 4 locations: A: Not reported R: 31%
Nguyen [51] (Singapore)	Improvement of 20 street segments (4.8 km in total) to complete a well-developed cycling network	Yes	20 intervention street segments vs 55 control street segments	Direct observation Time between measurements; 2 years Time exposed; 12 months	Bikes per hour	Difference-in-difference, but no statistical test conducted	Not reported	In favor of the intervention, significance not tested Average of the 20 locations: A: 18 bikes/hour R: 62%
Parker [52] (US)	Introduction of a bike lane (5.0 km) with multiple bus stops, schools, businesses, a police station and private residences located along the intervention	No	1 location on the study road	Direct observation Time between measurements; 12 months Time exposed; 6 months	Bikes per day	Difference tested by regression models	Not reported	In favor of the intervention, significant A: 53 bikes/day R: 58%
Parker [53] (US)	Introduction of a bike lane (1.6 km) with multiple schools, churches and businesses located along the intervention	Yes	1 location on the study road vs 1 location at 2 control streets	Direct observation Time between measurements; 12 months Time exposed; 3 months	Bikes per day	Difference-in-difference, but no statistical test conducted	Not reported	In favor of the intervention, significant A: 196 bikes/day R: 385%
Wilmink and Hartman [42] (The Netherlands)	Improvements to an existing cycle route network, creating a comprehensive and interconnected network	Yes	Counts made along roads in the intervention neighborhoods vs counts made in the control neighborhood	Count data, methods not described Time between measurements; 3 years Time exposed; not specified but could range from 1 to 3 years	Bike counts	Difference-in-difference, no statistical test conducted	Not reported	In favor of the intervention, significance not tested A: Not reported R: 14%

### Study results

**Fig. 2:**
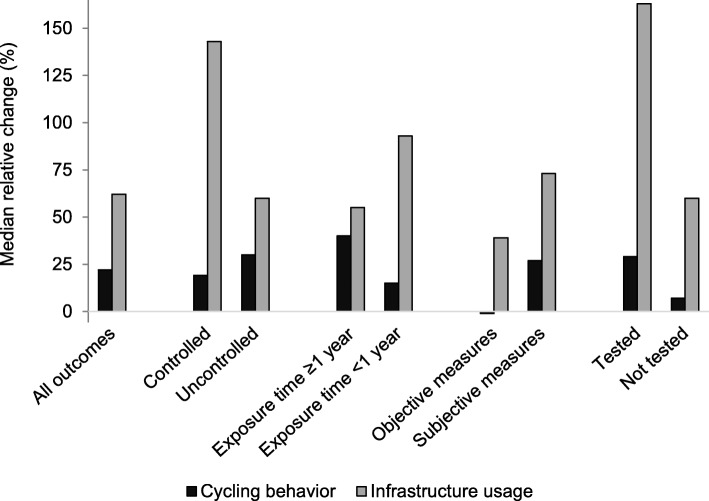
Summary of the results

Figure 2 presents an overview of median relative change for all outcomes reported, and according to study design, exposure time, method of assessment, and whether significance was tested. In general, studies reporting behavioral outcomes found smaller changes than studies presenting usage of the infrastructure. Larger changes were also found for studies that tested for statistical significance and studies that used subjective measurement methods (such as surveys and direct observations of cyclists), compared to studies that did not perform statistical tests, and used objective measurement methods (such as GPS and accelerometers, and automatic counting stations).

Additional file 4: Table S1 provides further details of the number of studies which assessed cycling behavior or usage of the infrastructure for cycling, and whether these were in favor of the intervention or not. Twenty studies presented data on 52 cycling behavior outcomes. All but two [23, 32], found an increase in cycling for at least 1 outcome, and 73% (38/52) of all outcomes presented were in favor of the intervention. A total of 36 cycling behavior outcomes were used to quantitatively summarize the results. Together, studies found a median relative increase in cycling behavior (median relative change: 23%; range: − 21 to 262%). Changes in cycling did not essentially differ between controlled and uncontrolled studies. Studies with an exposure time shorter than 1 year found smaller changes when compared to those using a longer exposure time. Studies that used objective measures to assess cycling behavior found smaller changes than those that used self-reported measures, and studies that did not test for statistical significance found smaller changes than those that did.

Seven studies evaluated changes in physical activity patterns following cycling infrastructure interventions. Brown et al. showed that among cyclists, cycling time on intervention streets increased by 7 min/week and on other streets increased by 6 min/week. Daily energy expenditure increased in the study population by 0.19 kcal/min, which translates into 275 kcal/day [26]. Goodman et al. found that living 1 km closer to the intervention increased cycling for recreation by 3 min/week, and total physical activity by 13 min/week [33]. There was no evidence that compensation of physical activity behaviors took place, since physical activity excluding walking and cycling was not associated with the intervention. Burbidge et al. did not find changes in total physical activity time, but the number physical activity episodes seemed to have declined by 0.2 trips/day following the introduction of cycling infrastructure [27]. The other four studies did not find evidence that the introduction of cycling infrastructure affected physical activity [29, 31, 32, 39].

Usage of the infrastructure was presented in 16 studies with 21 outcomes, and all were in favor of the intervention (median relative change: 62%; range: 4 to 438%) (Table 2). Changes for infrastructure usage were smaller for studies that were uncontrolled, studies with longer exposure time, studies using automatic counters or GPS tracking information, and studies that did not test for statistical significance (Additional file 4: Table S1).

**Table 2:** Description of the methodological quality, design elements and additional analyses

Reference (country)	Quality criteria [19]^a^						Methodological items [12]^b^	
	Study design^c^	Participation and representativeness	Comparability at baseline	Credibility of data collection methods	Retention	Attributability of effect to intervention	Multiple comparison groups	Complementing research methodologies
Cycling behavior								
Aittasalo [23] (Finland)	C	Participation: 49% Only limited data was available regarding the working-age population in the region. The study population was broadly representative with the general adult population in the region	No comparison group	Survey: no info on validity	45%	• Half of the workplaces went through economic problems and workforce adjustment during the study	No other comparison groups	• Published protocol • Survey among employees • Safety monitoring • Count data
Aldred [24] (UK)	A	Participation: 2% There was an underrepresentation of 16 to 24 year olds, non-white individuals and unemployed individuals. Participants were more likely to have a car or van in the household, and to have cycled	Comparisons groups were broadly comparable. Adjusted for a wide range of variables	7-day recall instrument with acceptable validity	50%	• Dose response effects were reported • The first interventions were targeting areas perceived as more receptive to cycling and walking interventions	No other comparison groups	• Survey among residents
Brown [25] (US)	A	Participation: 29% Representativeness was not shown	Adjusted for some of the characteristics in which the groups significantly differed at baseline	GPS and accelerometer data, using validated algorithm	59%	• Multiple improvements to other nearby infrastructure • Spill-over effect occurred: control residents were exposed to the intervention	No other comparison groups	• Published protocol • Survey among residents Health indicators: • Energy expenditure • BMI
Brown [26] (US)	C	Same as above	No comparison group	Same as above	Same as above	No comments made	No other comparison groups	Same as above
Burbidge and Goulias [27] (US)	C	Participation was not shown. Study population was older, had less cars in the household and were more often unemployed	No comparison group	1-day activity diary, modified from a validated household activity diary	56%	No comments made	No other comparison groups	• Survey among residents and new residents • Intercept survey Health indicators: • Physical activity
Chowdhury [28] (New Zealand)	C	Participation was not shown. Study population was representative	No comparison group	Survey, methods not described	Not applicable	No comments made	No other comparison groups	• Survey among residents
Crane [29] (Australia)	A	Participation was not shown. Study population was higher educated and more physically active than the general population	Adjusted for some of the characteristics in which the groups significantly differed	Survey: no info on validity Travel diary: no info on validity	48%	• No dose response effects were observed • Suburbs furthest away from the cycle way were quite diverse in infrastructure • Spill-over effect occurred: users of the cycle way included participants living in control areas	No other comparison groups	• Published protocol • Survey among residents • Count data Health indicators: • Physical activity • Quality of life
Deegan [30] (UK)	C	Participation and representativeness were not shown	No comparison group	Census data, methods not described	Not applicable	• Congestion charge and bombings on public transport resulted in sharp increases in cycling levels	• The increase in cycling in the intervention areas was larger than observed in other areas	• Safety monitoring
Dill [31] (US)	A	Participation: 3% Representativeness was not shown	Adjusted for variables that were tested to be significant	GPS and accelerometer data, shown to successfully predict 79% of the cycling trips	72%	• The city may have chosen to install bicycle boulevards in areas where residents were supportive of new cycling infrastructure • Unknown changes in the physical and social environment in specific areas may have influenced the results • Data collection by means of GPS and accelerometers may have changed behavior	No other comparison groups	• Survey among residents • Count data Health indicators: • Physical activity
Evenson [32] (US)	C	Participation: 47% Study population was more highly educated	No comparison group	Non validated method of interviewing	64%	• Questions mentioning the trail were only asked at follow-up and after assessing cycling behavior • Substitution of physical activity behavior may have occurred	• Comparing users and non-users of the intervention did not change the results	• Survey among residents Health indicators: • Physical activity
Goodman [33] (UK)	A	Participation: 16% Study population was broadly representative, except that fewer young adults were included, and they were somewhat healthier, better educated, and less likely to have children	Adjusted for a wide range of variables	7-day recall instrument with acceptable validity Survey, validated	42%	• Dose response effects were reported • The increase in cycling was only seen for users of the intervention	• Comparing users and non-users of the intervention showed that the increase in cycling was only seen for users of the intervention	• Published protocol • Survey among residents Health indicators: • Physical activity
Song [34] (UK)	C	Same as above	No comparison group	7-day recall instrument with acceptable validity Survey, validated	45%	• The increase in cycling may have resulted from the economic crisis, rising fuel costs, and the ageing of the sample	No other comparison groups	Same as above
Hirsch [35] (US)	A	Participation and representativeness were not shown.	Adjusted for a wide range of variables	Census data (before) and a community survey (after): no info on validity	Not applicable	• Dose response effects were reported • Unknown if people moving into the neighborhood cycle more, or if existing residents change their behaviors • Other infrastructure changes, including a new light rail service, may have influenced the results	• Historical trends showed that the increase in cycling in the intervention period was larger than in previous years	No other methods used
Krizek [36] (US)	C	Participation and representativeness were not shown.	No comparison group	Census data, methods not described	Not applicable	Many potential factors were listed, but only those with an explanation were listed here: • Minor other infrastructural improvements were made in the study areas • Small demographic differences were not the sole explanation of the results • Intervention areas had already a higher cycling level at baseline. The facilities might be the effect, rather than the cause, of high cycling levels	• The increase in cycling in the intervention area was larger than observed in the area as a whole	No other methods used
Lanzendorf [37] (Germany)	C	Participation and representativeness were not shown.	No comparison group	National travel survey, valid for comparison over time according to the authors	Not applicable	• Hard to disentangle the effects of the infrastructure and marketing campaigns. A combination of both may have the largest impact	• The increase in cycling in the intervention cities was comparable to the change in other big cities, but larger than in the country as a whole	• Document analysis and expert interview to analyze the development of cycling policies
Merom [38] (US)	A	Participation: 48% Representativeness was not shown.	Not adjusted for characteristics in which the groups statistically differed at baseline	Telephone interviews, validated	79%	No comments made	No other comparison groups	• Survey among residents • Bike counts • Campaign reach
Panter [39] (UK)	A	Participation was not shown. The sample contained a higher percentage of woman, older adults and those with a degree, and a smaller proportion of those who rented their home	Adjusted for a wide range of variables	7-day recall instrument with acceptable validity Survey, validated	41%	• Dose response effects were reported	No other comparison groups	• Published protocol • Survey among residents Health indicators: • Physical activity
Pedroso [40] (US)	B	Participation and representativeness were not shown	No comparison group	Census data, methods not described	Not applicable	• Several other programs and interventions were implemented during the study period	No other comparison groups	• Safety monitoring
Smith [41] (US)	C	Participation and representativeness were not shown	No comparison group	Methods not described	Not applicable	• The percentage of cyclists using bike lanes declined over time	No other comparison groups	• Safety monitoring
Wilmink and Hartman [42] (The Netherlands)	A	Participation and representativeness were not shown	No information on comparability	Home interview, no info on validity	Not shown	• There was no change observed in total mobility over time	No other comparison groups	• Survey among residents • Bike counts
Usage of the infrastructure								
Aittasalo [23] (Finland)	C	Not applicable	No comparison group	Automatic counters: 4 locations, continuous measurements for 2 years	Not applicable	• Half of the workplaces went through economic problems and workforce adjustment during the study	No other comparison groups	• Published protocol • Survey among employees • Safety monitoring • Cycling behavior
Barnes [43] (US)	C	Not applicable	No comparison group	54 h of video observations: before and after at 1 location, 6 days, 4.5 h per day	Not applicable	• No unusual weather or traffic patterns were observed • It is unclear whether cyclist simply changed their routes	No other comparison groups	• Safety monitoring
Crane [29] (Australia)	B	Not applicable	No comparison group	Automatic counters: 2 locations, measurements in October for 3 years on weekdays, 6 h per day	Not applicable	• Results may reflect population growth	• The increase in cyclists was only seen in the intervention area, while it decreased in the city as a whole • Historical trends in the number of cyclists were comparable between the intervention areas and the city as a whole	• Published protocol • Survey among residents • Cycling behavior Health indicators: • Physical activity • Quality of life
Dill [31] (US)	C	Not applicable	No comparison group	Not described in the paper	Not applicable	• Unknown changes in the physical and social environment in specific areas	No other comparison groups	• Survey among residents • Cycling behavior Health indicators: • Physical activity
Fitzhugh [44] (US)	A	Not applicable	Broadly comparable	72 h of direct observations: before and after at 3 locations, 2 days, 6 h per day	Not applicable	• Study neighborhoods were not exposed to any marketing or awareness campaigns • Spill-over effect may have occurred: people cycling may not live in the intervention neighborhood	No other comparison groups	No other methods used
Goodno [45] (US)	C	Not applicable	No comparison group	Not described in the paper	Not applicable	• Weather conditions and seasonality may have influenced the results	• The increase in cyclists in the intervention area was larger than in the city as a whole	• Survey among residents • Survey among business owners • Safety monitoring • Intercept survey
Hans [46] (Denmark)	B	Not applicable	No comparison group	Automatic counters calibrated by visual/manual counts: 2 locations, continuous measurements for 3 years	Not applicable	• Most of the increase in cyclists can be attributed to switching from alternative routes	No other comparison groups	• Intercept survey
Heesch [47] (Australia) - direct observations	C	Not applicable	No comparison group	7.5 h of direct observations: Before: 1 location, 1 day, 2.5 h After: 2 locations, 1 day, 2.5 h	Not applicable	• The findings suggest some shifting of cyclist	No other comparison groups	• Intercept survey • Mobile phone application to capture movements of cyclists
Heesch [47] (Australia) - mobile phone application	A	Only 10% of the population uses the app, and those were not representative of the broader cycling community	Comparison streets were all connecting the suburbs and the city center	1 year counts made by a mobile phone application: 4 locations, continuous measurement for 1 year	Not applicable	• The findings suggest some shifting of cyclist • The increase in people using the app may have influenced the results • Data on trips was analyzed, and it is unknown if the same cyclists were travelling more frequent, or if more cyclists were travelling	No other comparison groups	• Intercept survey • Direct observations
Law [48] (UK)	C	Not applicable	No comparison group	Before: direct observations: 21 locations, 1 day, 10 hAfter: automatic counters: 21 locations, 1 day,12 h	Not applicable	• The intervention effect is likely to be over-estimated due to seasonal differences • Change in data collection methods may have influenced the results	No other comparison groups	• Safety monitoring
Marques [49] (Spain)	B	Not applicable	No comparison group	Counts data, no description of the protocol	Not applicable	• Changes in population were not meaningful • Changes in data procedures over time may have influenced the results	No other comparison groups	• Safety monitoring
McCartney [50] (UK)	B	Not applicable	No comparison group	560 h of digital video recordings manually checked: before and after at 5 locations, 4 days, 14 h	Not applicable	• Displacement effects were observed • Weather and seasonality may have influenced the results • Traffic conditions may have influenced the results	• The relative increase in cyclists in the intervention area was larger than in the city as a whole	No other methods used
Merom [38] (US)	B	Not applicable	No comparison group	Automatic counters: 4 locations, continuous measurements for 5 months	Not applicable	No comments made	No other comparison groups	• Survey among residents • Cycling behavior • Campaign reach
Nguyen [51] (Singapore)	A	Not applicable	No information on comparability	Direct observations: few weekdays during peak periods, no precise description of the protocol	Not applicable	• Shifting of routes was observed • No major change in land use • Possibly reverse causation since segments that were improved had a high demand before the intervention	• The increase in cyclists was even larger on segments that were already improved before start of the current study	• Survey among residents • Intercept survey
Parker [52] (US)	C	Not applicable	No comparison group	216 h of direct observations; Before: 1 location, 10 days, 9 h After: 1 location, 14 days, 9 h	Not applicable	• Displacement from other streets may have occurred • It is possible that more people ride a bike because of the rising costs of car ownership • The population increase may have influenced the results, but it is unlikely that this explains the total change in cycling	No other comparison groups	No other methods used
Parker [53] (US)	A	Not applicable	No information on comparability	660 h of direct observations: before and after at 3 location, 10 days, 11 h	Not applicable	• Some displacement of cyclists from nearby streets was observed • Change in population size is unlikely to be the reason for the increase in cycling	No other comparison groups	No other methods used
Wilmink and Hartman [42] (The Netherlands)	A	Not applicable	No comparison group	Count data, no description of the protocol: 250 locations	Not applicable	• Population growth may have influenced the findings	No other comparison groups	• Survey among residents • Cycling behavior

^a^None of the studies was a randomized experiment, therefore randomization was not applicable for any of the studies and was not shown.

^b^None of the studies presented data for neutral outcomes that were hypothesized to be unaffected by the new infrastructure designed to promote cycling, therefore this parameter was not shown.

^c^A = controlled before-after study; B = uncontrolled study with at least two before and two after data points; C = uncontrolled study with only 1 before and after data point

### Quality assessment

Table 2 presents information on the quality of the studies. Nine out of twenty studies evaluating the impact of cycling infrastructure on cycling behavior presented data on participation, and nine on representativeness. Participation ranged between 2 and 49% for those that presented information. Thirteen studies collected data twice on the same individual, and retention ranged between 41 and 79%. Most studies used surveys to collect data, but the exact methodology and validity of the question items was often not reported.

When considering the quality of the studies for causal inference, studies reported that other changes in the physical and social environment might have affected or biased their results. Issues reported were the economic crisis, the rising cost of car transport, social marketing campaigns, and other infrastructural improvements during the same period. Authors were often unable to account for these and this could indicate that the changes observed could be partly attributable to other factors. Another problem mentioned is a spill-over effect, indicating that people from control areas might have used the facilities, which may have resulted in an underestimation of the effect. Some studies used multiple groups to test robustness of the findings by using different comparisons group or applying different cut-off values to define exposure or outcome. Some studies presented data for city- or nation-wide cycling trends [36, 37], or historical time trends [35]. None of the studies included a neutral outcome which was hypothesized to be unaffected by the new infrastructure designed to promote cycling, thereby functioning as a control measure that captures time trends in transportation or physical activity behaviors. Complementing methodologies performed were surveys among residents [24–29, 31–34, 38, 39, 42] or employees [23], intercept surveys among infrastructure users [27], surveys among new residents who moved into the study area [27], and bike counts in the study area [23, 29, 31, 38, 42].

Sixteen studies presented data on usage of the infrastructure. Five studies used automatic counting stations or mobile app data to objectively measure cyclist movements for periods between 5 months and 3 years. Others monitored the number of cyclist on selected hours and days using observation techniques. Issues that authors reported that may have partly contributed to the increase in infrastructure usage were tunneling of existing riders to the new infrastructure, other infrastructural changes, traffic conditions, rising cost of car transport, weather conditions and seasonality, demographic changes, social marketing, and changing methodology to collect data. One study indicated that improvements made to the cycling infrastructure could have been a consequence of high cycling levels in specific areas [51]. Some studies presented data for city- or nation-wide cycling trends [29, 45, 50], or historical time trends [29]. Additional methodologies included surveys among residents [31, 38, 42, 45, 50, 51] or employees [23], survey among infrastructure users [45–47, 51], and data collected on cycling behavior [23, 29, 31, 38, 42].

### Equity effects

**Fig. 3:**
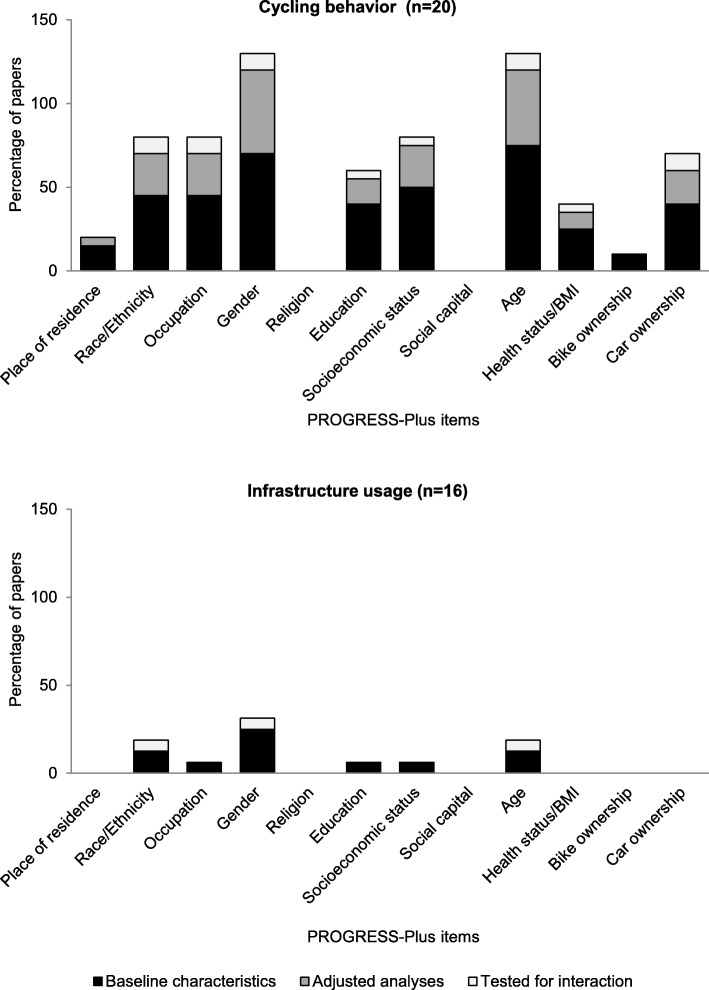
Percentage of studies that presented equity characteristics from the PROGRESS-Plus framework

Figure 3 shows that studies assessing cycling behavior collected information on population characteristics more often than those assessing usage, thereby potentially providing insights in the population under study and characteristics of those engaging in cycling, and allowing a comparison of intervention and control groups according to baseline characteristics. The items that were most often used by behavioral studies to describe the population at baseline were age (75%), gender (70%) and a measure of socio-economic status (SES) (50%). Only three studies tested for differential effects on cycling by population subgroups. Aldred et al. did not find any differential effects by demographic and socio-economic characteristics [24]. Goodman et al. showed that the change in cycling behavior was larger if there was no car in the household [33]. Parker et al. showed that the increase in cyclists was larger among females than males [53].

## Discussion

We identified 31 studies that assessed the effect of infrastructural interventions on cycling in adult populations. All were conducted in urban areas in high-income countries. Most of the evaluations found effects in favor of the intervention, showing that the number of cyclists using the facilities increased, and to a lesser extent that cycling behavior increased. Studies that collected behavioral data more often provided insights in characteristics of people engaging in cycling as compared to studies that reported bike counts. Seven studies reported on physical activity levels, and findings were mixed. Only three studies tested for equity effects, therefore we cannot draw any conclusions as to whether some population subgroups benefitted more than others. We provided data on relative changes that indicates the magnitude of the findings. We acknowledge that in context where only few people use a bike, large relative changes may result in only small population-health benefits. However, due to the large variety in outcomes used we could not further summarize the results.

Our findings suggest that the approach and the specific methods did provide different results. Previous reviews have indicated that this might be the case, but our synthesis of studies exclusively focusing on cycling according to the method used, provides more evidence of this [15, 16]. This review built on earlier findings by including studies with various study designs and published in health-related and transportation-related journals. Furthermore, we quantitatively summarized the findings to assess whether the magnitude of the change in cycling differed across study design. In the following three sections we describe the implications of the study design, data collection methods and statistical approaches for the study findings.

### Study design and implications for causal inference

An important aspect of study design is the choice of outcome. In this review we categorized outcomes broadly into those that assessed cycling behavior and infrastructure usage. We found that studies on behavioral outcomes found smaller relative changes than studies presenting usage of the infrastructure. If researchers are interested in outcomes relevant for population health, it is recommended that outcomes are framed around the duration and frequency of cycling, as these measures can be directly linked to health impacts. Assessing the proportion of cyclists in a population or the numbers using a route may be a good alternative. If researchers are interested in understanding usage, count data may be used to measure the number of cyclists on the new infrastructure. Other reviews also found that studies measuring outcomes more closely related to the intervention (for example: cycling) were more likely to find intervention effects than studies measuring more general outcomes (for example: physical activity or BMI) [15, 54]. Bike count data may support the findings from other evaluations on cycling behavior, but it cannot directly be translated into health gains in the population.

Another important design element is whether to include a control population when evaluating built environment changes. The changes in cycling differed for controlled and uncontrolled studies that assessed usage of the infrastructure, but not for cycling behavior. Uncontrolled studies have a stronger basis for causal inference if they can provide evidence that the observed effects do not solely reflect underlying time trends in cycling in the wider area [29, 36, 37, 45, 50]. For example, Crane [29] counted the number of bikes passing 2 locations along the new infrastructure. They also presented city-wide cycling trends during the same time period. An increase of 3.7% of cyclist was found along the intervention road, whereas a decrease of 2.0% was seen in the city as a whole. This finding suggests that the number of cyclist increased in the area with the new infrastructure, and this increase does not solely reflect underlying time trends in cycling. To strengthen causal inference, we recommend that studies use controlled designs where possible, and present different measures of cycling and physical activity. Evaluating similar interventions across different sites could give further insights in the variation in the change in these sites if controlled designs are not possible. For example, Lanzendorf [37] evaluated improvements made to the cycling infrastructure in 4 German cities. Cycling frequency on average increased by 27%, which differed between cities from 3 to 38%. They also reported an average increase of cycling frequency by 31% in all big German cities. This approach illustrates that the observed changes in cycling in the intervention sites were comparable to the country-wide increase in cycling. The large range in changes in cycling in the 4 intervention sites also gives insight into the potential range of effects which could be expected in other cities.

The duration of time that populations are exposed to the new infrastructure is another important design element, which can be difficult to control in large infrastructural projects. In studies that assessed changes in cycling behavior we found that the changes were larger when exposure time was longer than 1 year. In studies that assessed the usage of cycling infrastructure, those with shorter exposure time reported larger changes than those with longer exposure time. We noted that some count studies did not count on rainy days [44, 53], or only collected data during peak hours [23, 29, 45, 47, 51], which may have resulted in larger changes than what could be expected if data was measured throughout by means of automatic counters [46]. Most studies that found changes that were not in favor of the intervention were less than 6 months exposed [23, 25, 31, 32], suggesting that longer follow-up periods may be needed to allow behavioral changes to be detected. Including questions on infrastructure usage within ongoing surveys, or nested within cohorts, may ensure that if the construction work is delayed, there is data available with sufficient exposure time to measure the impact.

### Data collection methods and implications for causal inference

Studies were categorized according to whether the focus was on usage or cycling behavior, and large differences in results were found between these two types of outcome. Studies presenting count data of infrastructure found larger changes than studies that assessed behavioral change in the population. Studies counting the number of bikes that passed tracking locations are at risk of assessing the displacement of existing riders to the new infrastructure, and seven studies specifically mentioned this phenomena [43, 46, 47, 50–53]. Some studies had offset some of the so-called funneling biases by selecting strategic counting locations where most cyclist pass, or used multiple counting locations to capture cycling behavior in a wider area. Some studies complemented bike count data with intercept surveys among users of the infrastructure, and asked about their previous travel behaviors. These studies showed that the proportion of users that would not have cycled, had the infrastructural improvement not taken place, was much smaller than the increase in counts of cyclists [46, 47, 51]. Bike count data is useful when aiming to describe at what times of the day, and under which weather conditions, cyclists are using the facility [46].

Another important consideration is choosing between objective or self-reported measures to collect data on cycling behavior. We found that studies using GPS and other objective measures of cycling reported smaller changes than those using self-reported measures. Using GPS and objective assessments of activity could potentially be used to distinguish cycling on and off the new infrastructure [26], and yields estimates of total physical activity levels [26, 31]. However, such measures are often applied to a small sample, are limited to a short period of time, and participants who wear such devices might be quite different to the general population. Therefore the findings might be subject to some selection biases. Furthermore, it is possible that the novelty of wearing such devices might lead to changes in physical activity behaviors [31]. Subjective measures of cycling behaviors, such as travel diaries and surveys, provide alternatives when interested in larger groups of people, but many of these have not been validated for cycling specifically.

It is attractive to use already available data when studying so-called “natural experiments” in which researchers lack control over the intervention. Collecting new data to match the timescale of intervention delivery is challenging. A third of the studies evaluating cycling behavior used data that were already collected for a regular monitoring or as part of other studies for the evaluation of other built environment interventions [28, 30, 35–37, 40, 41]. For example, four US studies used census data to estimate changes in cycling after the introduction of new cycling facilities [35, 36, 40, 41]. Other evaluations of natural experiments were planned, allowing to collect specific data to evaluate the intervention of interest in detail. This resulted in powerful analyses in which the method of data collection was tailored to the research questions, but sometimes resulted in limited time being exposed to the intervention. For example, Dill [31] assessed cycling at baseline and after 2-years of follow-up. The construction work was significantly delayed, resulting in a short time period between the opening of the facilities and the second assessment of cycling. Moreover, two of the nine projects were not completed within this period. This may have influenced study outcomes. Using existing data may be useful if researchers were not aware of the new intervention, did not obtain funding in time to design a study around the natural experiment, or if large delays in the construction are expected.

### Analytical approaches and implications for causal inference

Like other reviews [55], we found that many studies did not perform statistical tests (for cycling behavior: 15% (8/52) and for usage: 67% (14/21)). Smaller changes were found for studies that did not test for statistical significance than those that performed statistical tests. We recommend that studies test for statistical significance which provides more robust evidence that the results are not due to chance, as recommended by guidance for the clear reporting of observational studies [56]. This review included some studies that used more complex analytical methods, such as fixed-effect models [27], interrupted time series [47], or estimated the difference in cycling over time by using a regression analyses that included group, period, and an interaction term between group and period [29, 31, 35]. Fixed-effects models allow to account for observed time-varying and unobserved time-invariant characteristics. Perhaps most prominently, individual attitudes towards physical activity may both determine living at a place with opportunities to be physically active and their physical activity behavior. Fixed-effect models allow to control for such unobserved time-invariant confounding, allowing for better causal inference. One study conducted a time series analyses by using GPS tracking information from a mobile phone application, thereby correcting for time trends prior to the intervention [47]. Studies that specified an interaction term between group and period are able to control for observed differences between groups, thereby reducing the risk of bias. The usage of multiple analytical strategies, and the usage of methods that are able to correct for time trends, and measured or unmeasured confounders at the individual or neighborhood level may strengthening the basis of causal inference.

### Strengths and limitations

In this review, we focused on the methodological aspects in the evaluation of infrastructural interventions to promote cycling and extracted information on the magnitude of the change in cycling. This allowed us to examine differences in change in cycling according to the methods used. This study was comprehensive by searching multiple electronic databases without date or language restrictions, and we included studies published in public health journals and transportation journals. Controlled and uncontrolled studies were considered for inclusion, and the final selection of studies had a large variety in study designs and methods. We added valuable information by calculating the relative and absolute changes in cycling behavior or usage of the infrastructure, which brought together different outcomes in a simple but interpretable way.

Some limitations also have to be noted. We included only studies that reported on measures of cycling and were unable to examine unreported data on cycling that were included in composite measures of active transportation, walking and cycling, or physical activity. The detail of the information provided in the papers differed between studies, which made it difficult to synthesise and interpret study findings. A pragmatic approach was used to calculate relative changes where possible, but for some studies other approaches may have been better. The evidence presented in the review came from studies that were all conducted in high-income countries. Moreover, only a few studies evaluated the impact on physical activity behaviors and studied equity effects. We focused on structural interventions here, but future research should explore the importance of and interactions with other interventions, such as financial incentives, cycle training, or behavioral interventions, together with the introduction and maintenance of high-quality cycling infrastructure.

### Recommendations

Each study design, data collection method and analytical strategy has its advantages and disadvantages. To further strengthen causal inference from observational data, studies are needed that triangulate different methodologies to evaluate the effect of built environment interventions. Studies published in public health journals often report on changes in cycling behavior, while studies published in transportation journals report on usage of cycling infrastructure. Bringing experts from both fields together could result in study designs that better capture the range of impacts of new cycling infrastructure. We are not recommending a specific method or approach, as the research questions of interest should drive the method of data collection. When existing data are used, careful consideration needs to be given to the appropriateness of that data. The reporting of evaluations should adhere to guidelines, such as STROBE which seeks to strengthen the quality of work reported [56]. We suggest, where possible, to combine count data that provides information on how many people are using new infrastructure, with behavioral outcomes of duration and frequency of cycling to ensure estimates of the population health impact. Such estimates could be used in combination with modelling or scenario building tools to estimate the current or future health impacts on outcomes that cannot be observed in studies with limited follow-up. Future studies should focus on the question who are benefiting from the intervention, and identify contexts, barriers and choice constraints to better understanding why cycling changed. This review focused on interventions that changed the cycling infrastructure, but findings and recommendations are likely applicable to other built environment interventions to promote health behaviors.

## Conclusion

Introducing cycling facilities in cities is likely to increase the number of cyclist using the facilities, and may result in increases in cycling. Evidence on total physical activity following cycling facilities was mixed. Equity effects were rarely studied. Research questions interest should drive the method of data collection and reporting of evaluations should adhere to published guidelines. Triangulation of methods is warranted to overcome potential issues that evaluators may encounter when evaluating infrastructural interventions within the built environment, and to strengthen the basis of causal inference.

## Supplementary information

**Additional file 1**: Appendix 1. Search strategy.

**Additional file 2**: Appendix 2. Calculations of relative and absolute change.

**Additional file 3**: Appendix 3. Selection of articles.

**Additional file 4: Table S1**. Summary of the results.

## Data Availability

No additional data available.

## Abbreviations

RCT: Randomized Controlled Trial
SES: Socio-economic status

## References

[1] Kyu HH, Bachman VF, Alexander LT, Mumford JE, Afshin A, Estep K (2016). Physical activity and risk of breast cancer, colon cancer, diabetes, ischemic heart disease, and ischemic stroke events: systematic review and dose-response meta-analysis for the global burden of disease study 2013. Bmj.

[2] Oja P, Titze S, Bauman A, de Geus B, Krenn P, Reger-Nash B (2011). Health benefits of cycling: a systematic review. Scand J Med Sci Sports.

[3] Fishman E, Böcker L, Helbich M (2015). Adult active transport in the Netherlands: an analysis of its contribution to physical activity requirements. PLoS One.

[4] Kelly P, Kahlmeier S, Götschi T, Orsini N, Richards J, Roberts N (2014). Systematic review and meta-analysis of reduction in all-cause mortality from walking and cycling and shape of dose response relationship. Int J Behav Nutr Phys Act.

[5] Rasmussen MG, Grøntved A, Blond K, Overvad K, Tjønneland A, Jensen MK (2016). Associations between recreational and commuter cycling, changes in cycling, and type 2 diabetes risk: a cohort study of Danish men and women. PLoS Med.

[6] Blond K, Jensen MK, Rasmussen MG, Overvad K, Tjønneland A, Østergaard L (2016). Prospective study of bicycling and risk of coronary heart disease in Danish men and women. Circulation.

[7] de Hartog JJ, Boogaard H, Nijland H, Hoek G (2010). Do the health benefits of cycling outweigh the risks?. Environ Health Perspect.

[8] Tainio M, de Nazelle AJ, Götschi T, Kahlmeier S, Rojas-Rueda D, Nieuwenhuijsen MJ (2016). Can air pollution negate the health benefits of cycling and walking?. Prev Med.

[9] Pucher J, Buehler R (2008). Making cycling irresistible: lessons from The Netherlands, Denmark and Germany. Transp Rev.

[10] Garrard J, Rose G, Lo SK (2008). Promoting transportation cycling for women: The role of bicycle infrastructure. Prev Med.

[11] Dill J (2009). Bicycling for transportation and health: The role of infrastructure. J Public Health Policy.

[12] Craig P, Cooper C, Gunnell D, Haw S, Lawson K, Macintyre S (2012). Using natural experiments to evaluate population health interventions: new Medical Research Council guidance. J Epidemiol Community Health.

[13] Craig P, Katikireddi SV, Leyland A, Popham F (2017). Natural experiments: an overview of methods, approaches, and contributions to public health intervention research. Annu Rev Public Health.

[14] Barnighausen T, Tugwell P, Rottingen JA, Shemilt I, Rockers P, Geldsetzer P (2017). Quasi-experimental study designs series - paper 4: uses and value. J Clin Epidemiol.

[15] Stappers NEH, Van Kann DHH, Ettema D, De Vries NK, Kremers SPJ (2018). The effect of infrastructural changes in the built environment on physical activity, active transportation and sedentary behavior – a systematic review. Health & Place.

[16] Panter J, Guell C, Humphreys D, Ogilvie D (2019). Can changing the physical environment promote walking and cycling? A systematic review of what works and how. Health Place.

[17] Moher D, Liberati A, Tetzlaff J, Altman DG, The PG (2009). Preferred reporting items for systematic reviews and meta-analyses: The PRISMA statement. PLoS Med.

[18] Goodman A, Panter J, Sharp SJ, Ogilvie D (2013). Effectiveness and equity impacts of town-wide cycling initiatives in England: a longitudinal, controlled natural experimental study. Soc Sci Med.

[19] Ogilvie D, Fayter D, Petticrew M, Sowden A, Thomas S, Whitehead M (2008). The harvest plot: a method for synthesising evidence about the differential effects of interventions. BMC Med Res Methodol.

[20] Briss PA, Zaza S, Pappaioanou M, Fielding J, Wright-De Agüero L, Truman BI (2000). Developing an evidence-based guide to community preventive services—methods. Am J Prev Med.

[21] Thomas H (2003). Quality assessment tool for quantitative studies.

[22] O'Neill J, Tabish H, Welch V, Petticrew M, Pottie K, Clarke M (2014). Applying an equity lens to interventions: using PROGRESS ensures consideration of socially stratifying factors to illuminate inequities in health. J Clin Epidemiol.

[23] Aittasalo Minna, Tiilikainen Johanna, Tokola Kari, Suni Jaana, Sievänen Harri, Vähä-Ypyä Henri, Vasankari Tommi, Seimelä Timo, Metsäpuro Pasi, Foster Charlie, Titze Sylvia (2019). Socio-Ecological Natural Experiment with Randomized Controlled Trial to Promote Active Commuting to Work: Process Evaluation, Behavioral Impacts, and Changes in the Use and Quality of Walking and Cycling Paths. International Journal of Environmental Research and Public Health.

[24] Aldred R, Croft J, Goodman A (2019). Impacts of an active travel intervention with a cycling focus in a suburban context: one-year findings from an evaluation of London's in-progress mini-Hollands programme. Transportation Res Part a-Policy Pract.

[25] Brown BB, Smith KR, Tharp D, Werner CM, Tribby CP, Miller HJ (2016). A complete street intervention for walking to transit, nontransit walking, and bicycling: a quasi-experimental demonstration of increased use. J Phys Act Health.

[26] Brown BB, Tharp D, Tribby CP, Smith KR, Miller HJ, Werner CM (2016). Changes in bicycling over time associated with a new bike lane: relations with kilocalories energy expenditure and body mass index. J Transp Health.

[27] Burbidge SK, Goulias KG (2009). Evaluating the impact of Neighborhood Trail development on active travel behavior and overall physical activity of suburban residents. Transp Res Rec.

[28] Chowdhury S, Costello SB (2016). An examination of cyclists' and non-cyclists' mode choice under a new cycle network. Road Transp Res.

[29] Crane M, Rissel C, Standen C, Ellison A, Ellison R, Wen LM (2017). Longitudinal evaluation of travel and health outcomes in relation to new bicycle infrastructure, Sydney, Australia. J Transp Health.

[30] Deegan B (2016). Cycling infrastructure in London.

[31] Dill J, McNeil N, Broach J, Ma L (2014). Bicycle boulevards and changes in physical activity and active transportation: findings from a natural experiment. Prev Med.

[32] Evenson KR, Herring AH, Huston SL (2005). Evaluating change in physical activity with the building of a multi-use trail. Am J Prev Med.

[33] Goodman A, Sahlqvist S, Ogilvie D (2014). iConnect C. new walking and cycling routes and increased physical activity: one- and 2-year findings from the UK iConnect study. Am J Public Health.

[34] Song Y, Preston J, Ogilvie D, iConnect c (2017). New walking and cycling infrastructure and modal shift in the UK: a quasi-experimental panel study. Transp Res Part A Policy Pract.

[35] Hirsch JA, Meyer KA, Peterson M, Le Z, Rodriguez DA, Gordon-Larsen P (2017). Municipal investment in off-road trails and changes in bicycle commuting in Minneapolis, Minnesota over 10 years: a longitudinal repeated cross-sectional study. Int J Behav Nutr Phys Act.

[36] Krizek KJ, Barnes G, Thompson K (2009). Analyzing the effect of bicycle facilities on commute mode share over time. J Urban Plann Dev.

[37] Lanzendorf M, Busch-Geertsema A (2014). The cycling boom in large German cities-empirical evidence for successful cycling campaigns. Transp Policy.

[38] Merom D, Bauman A, Vita P, Close G (2003). An environmental intervention to promote walking and cycling - The impact of a newly constructed Rail Trail in Western Sydney. Prev Med.

[39] Panter J, Heinen E, Mackett R, Ogilvie D (2016). Impact of new transport infrastructure on walking, cycling, and physical activity. Am J Prev Med.

[40] Pedroso FE, Angriman F, Bellows AL, Taylor K (2016). Bicycle use and cyclist safety following Boston's bicycle infrastructure expansion, 2009-2012. Am J Public Health.

[41] Smith A, Zucker S, Lladó-Farrulla M, Friedman J, Guidry C, McGrew P (2019). Bicycle lanes: are we running in circles or cycling in the right direction?. J Trauma Acute Care Surg.

[42] Wilmink A, Hartman JB (1987). Evaluation of the Delft bicycle network plan. Final Summary Report.

[43] Barnes E, Schlossberg M (2013). Improving cyclist and pedestrian environment while maintaining vehicle throughput before- and after-construction analysis. Transp Res Rec.

[44] Fitzhugh EC, Bassett DR, Evans MF (2010). Urban trails and physical activity: a natural experiment. Am J Prev Med.

[45] Goodno M, McNeil N, Parks J, Dock S (2013). Evaluation of innovative bicycle facilities in Washington, DC Pennsylvania avenue median lanes and 15th street cycle track. Transp Res Rec.

[46] Hans SP, Bredahl JJ, Elizabeth VS, Nielsen TAS, Simon R (2017). Effects of upgrading to cycle highways - an analysis of demand induction, use patterns and satisfaction before and after. J Transp Geogr.

[47] Heesch KC, James B, Washington TL, Zuniga K, Burke M (2016). Evaluation of the Veloway 1: a natural experiment of new bicycle infrastructure in Brisbane, Australia. J Transp Health.

[48] Law S, Sakr FL, Martinez M (2014). Measuring the changes in aggregate cycling patterns between 2003 and 2012 from a space syntax perspective. Behav Sci (Basel).

[49] Marqués R, Hernández-Herrador V (2017). On the effect of networks of cycle-tracks on the risk of cycling. The case of Seville. Accid Anal Prev.

[50] McCartney G, Whyte B, Livingston M, Crawford F (2012). Building a bridge, transport infrastructure and population characteristics: explaining active travel into Glasgow. Transp Policy.

[51] Nguyen PN, Koh PP, Wong YD (2015). Impacts of bicycle infrastructure: a case study in Singapore. Proc Inst Civil Eng-Munic Eng.

[52] Parker KM, Gustat J, Rice JC (2011). Installation of bicycle lanes and increased ridership in an urban, mixed-income setting in New Orleans, Louisiana. J Phys Act Health.

[53] Parker KM, Rice J, Gustat J, Ruley J, Spriggs A, Johnson C (2013). Effect of bike lane infrastructure improvements on ridership in one New Orleans neighborhood. Ann Behav Med.

[54] Mayne SL, Auchincloss AH, Michael YL (2015). Impact of policy and built environment changes on obesity-related outcomes: a systematic review of naturally occurring experiments. Obes Rev.

[55] Yang L, Sahlqvist S, McMinn A, Griffin SJ, Ogilvie D (2010). Interventions to promote cycling: systematic review. Bmj.

[56] von Elm E, Altman DG, Egger M, Pocock SJ, Gøtzsche PC, Vandenbroucke JP (2014). The strengthening the reporting of observational studies in epidemiology (STROBE) statement: guidelines for reporting observational studies. Int J Surg.

