# Supplementary material for: A systematic review of the effect of infrastructural interventions to promote cycling: strengthening causal inference from observational data
Source: Int J Behav Nutr Phys Act. 2019 Oct 26;16:93. doi: 10.1186/s12966-019-0850-1 (PMC6815350; doi:10.1186/s12966-019-0850-1)
Supplement: Supplementary file 1 — Additional file 1: Appendix 1. Search strategy. [file 12966_2019_850_MOESM1_ESM.docx]

**A systematic review of the effect of infrastructural interventions to promote cycling: Strengthening causal inference from observational data**

*Famke J.M. Mölenberg, Jenna Panter, Alex Burdorf, Frank J. van Lenthe*

**Appendix 1. Search strategy**

**embase.com**

(('environmental planning'/de AND ('cycling'/de OR 'bicycle'/de)) OR ((bicycle* OR cycling OR cyclist* OR biking OR bike ) NEAR/6 (lane* OR path* OR trail* OR infrastructure* OR planning OR design* OR network* OR space OR zone OR zones OR track* OR dedicate* OR roadway* OR boulevard* OR route*) OR Bikeabilit* OR transport-infrastructure* OR (Cycle NEXT/1 (lane OR path OR track OR lanes OR paths OR tracks OR network*)) OR (cycle NEAR/3 (infrastructure* OR dedicate* OR path OR paths)) OR ((bicycle* OR cycling OR cyclist* OR biking OR bike ) AND ((environment* OR infrastructur* OR neighbourhood* OR neighborhood* OR roadway* OR boulevard* OR route*) NEAR/6 (interven* OR change* OR plan* OR design* OR correlat* OR factor* OR determinant* OR difference* OR structure* OR characteristic* OR measure* OR built OR role OR condition* OR context* OR improve*)))):ab,ti) AND ('lifestyle modification'/de OR 'healthy lifestyle'/de OR 'physical activity'/de OR 'active transport'/de OR commuting/de OR 'leisure'/de OR 'recreation'/de OR 'behavior change'/de OR 'health promotion'/de OR 'motor activity'/de OR (((lifestyle OR life-style) NEAR/3 (modif* OR change* OR behav* OR healthy)) OR ((physical* OR transport* OR travel*) NEAR/3 (activ* OR inactiv*)) OR commut* OR leisur* OR recreat* OR ((transport* OR Prevalen* OR predictor* OR statistic* OR utilit* OR promot* OR increase* OR play OR use OR using) NEAR/6 (cycling OR bicycling OR cyclist* OR biking OR bike)) OR (behav* NEAR/3 (change* OR healthy)) OR (mode NEAR/3 travel) OR (health NEAR/3 promotion) OR (motor NEAR/3 activit*)):ab,ti) NOT ([Conference Abstract]/lim OR [Letter]/lim OR [Note]/lim OR [Editorial]/lim)

**Medline Ovid**

(((Environment Design/ OR Public Facilities/) AND (Bicycling/)) OR ((bicycle* OR cycling OR cyclist* OR biking OR bike ) ADJ6 (lane* OR path* OR trail* OR infrastructure* OR planning OR design* OR network* OR space OR zone OR zones OR track* OR dedicate*) OR Bikeabilit* OR transport-infrastructure* OR (Cycle ADJ (lane OR path OR track OR lanes OR paths OR tracks OR network*)) OR (cycle ADJ3 (infrastructure* OR dedicate* OR path OR paths)) OR ((bicycle* OR cycling OR cyclist* OR biking OR bike ) AND ((environment* OR infrastructur* OR neighbourhood* OR neighborhood* OR roadway* OR boulevard* OR route*) ADJ6 (interven* OR change* OR plan* OR design* OR correlat* OR factor* OR determinant* OR difference* OR structure* OR characteristic* OR measure* OR built OR role OR condition* OR context* OR improve*)))).ab,ti.) AND (Leisure Activities/ OR recreation/ OR health promotion/ OR motor activity/ OR (((lifestyle OR life-style) ADJ3 (modif* OR change* OR behav* OR healthy)) OR ((physical* OR transport* OR travel*) ADJ3 (activ* OR inactiv*)) OR commut* OR leisur* OR recreat* OR ((transport* OR Prevalen* OR predictor* OR statistic* OR utilit* OR promot* OR increase* OR play OR "use" OR using) ADJ6 (cycling OR bicycling OR cyclist* OR biking OR bike)) OR (behav* ADJ3 (change* OR healthy)) OR (mode ADJ3 travel) OR (health ADJ3 promotion) OR (motor ADJ3 activit*)).ab,ti.) NOT (letter OR news OR comment OR editorial OR congresses OR abstracts).pt.

**PsycINFO Ovid**

(((bicycle* OR cycling OR cyclist* OR biking OR bike ) ADJ6 (lane* OR path* OR infrastructure* OR planning OR design* OR network* OR space OR zone OR zones OR track* OR dedicate*) OR Bikeabilit* OR transport-infrastructure* OR (Cycle ADJ (lane OR path OR track OR lanes OR paths OR tracks OR network*)) OR (cycle ADJ3 (infrastructure* OR dedicate* OR path OR paths)) OR ((bicycle* OR cycling OR cyclist* OR biking OR bike ) AND ((environment* OR infrastructur* OR neighbourhood* OR neighborhood* OR roadway* OR boulevard* OR route*) ADJ6 (interven* OR change* OR plan* OR design* OR correlat* OR factor* OR determinant* OR difference* OR structure* OR characteristic* OR measure* OR built OR role OR condition* OR context* OR improve*)))).ab,ti.) AND (Leisure Time/ OR recreation/ OR health promotion/ OR (((lifestyle OR life-style) ADJ3 (modif* OR change* OR behav* OR healthy)) OR ((physical* OR transport* OR travel*) ADJ3 (activ* OR inactiv*)) OR commut* OR leisur* OR recreat* OR ((transport* OR Prevalen* OR predictor* OR statistic* OR utilit* OR promot* OR increase* OR play OR "use" OR using) ADJ6 (cycling OR bicycling OR cyclist* OR biking OR bike)) OR (behav* ADJ3 (change* OR healthy)) OR (mode ADJ3 travel) OR (health ADJ3 promotion) OR (motor ADJ3 activit*)).ab,ti.) NOT (letter OR news OR comment OR editorial OR congresses OR abstracts).pt.

**CINAHL EBSCOhost**

(TI ((bicycle* OR cycling OR cyclist* OR biking OR bike ) N5 (lane* OR path* OR infrastructure* OR planning OR design* OR network* OR space OR zone OR zones OR track* OR dedicate*) OR Bikeabilit* OR transport-infrastructure* OR (Cycle N1 (lane OR path OR track OR lanes OR paths OR tracks OR network*)) OR (cycle N2 (infrastructure* OR dedicate* OR path OR paths)) OR ((bicycle* OR cycling OR cyclist* OR biking OR bike ) AND ((environment* OR infrastructur* OR neighbourhood* OR neighborhood* OR roadway* OR boulevard* OR route*) N5 (interven* OR change* OR plan* OR design* OR correlat* OR factor* OR determinant* OR difference* OR structure* OR characteristic* OR measure* OR built OR role OR condition* OR context* OR improve*)))) OR AB ((bicycle* OR cycling OR cyclist* OR biking OR bike ) N5 (lane* OR path* OR infrastructure* OR planning OR design* OR network* OR space OR zone OR zones OR track* OR dedicate*) OR Bikeabilit* OR transport-infrastructure* OR (Cycle N1 (lane OR path OR track OR lanes OR paths OR tracks OR network*)) OR (cycle N2 (infrastructure* OR dedicate* OR path OR paths)) OR ((bicycle* OR cycling OR cyclist* OR biking OR bike ) AND ((environment* OR infrastructur* OR neighbourhood* OR neighborhood* OR roadway* OR boulevard* OR route*) N5 (interven* OR change* OR plan* OR design* OR correlat* OR factor* OR determinant* OR difference* OR structure* OR characteristic* OR measure* OR built OR role OR condition* OR context* OR improve*))))) AND (MH Leisure Activities OR MH recreation OR MH health promotion OR MH motor Activity OR (TI ((lifestyle OR life-style) N2 (modif* OR change* OR behav* OR healthy)) OR ((physical* OR transport* OR travel*) N2 (activ* OR inactiv*)) OR commut* OR leisur* OR recreat* OR ((transport* OR Prevalen* OR predictor* OR statistic* OR utilit* OR promot* OR increase* OR play OR "use" OR using) N5 (cycling OR bicycling OR cyclist* OR biking OR bike)) OR (behav* N2 (change* OR healthy)) OR (mode N2 travel) OR (health N2 promotion) OR (motor N2 activit*)) OR AB (((lifestyle OR life-style) N2 (modif* OR change* OR behav* OR healthy)) OR ((physical* OR transport* OR travel*) N2 (activ* OR inactiv*)) OR commut* OR leisur* OR recreat* OR ((transport* OR Prevalen* OR predictor* OR statistic* OR utilit* OR promot* OR increase* OR play OR "use" OR using) N5 (cycling OR bicycling OR cyclist* OR biking OR bike)) OR (behav* N2 (change* OR healthy)) OR (mode N2 travel) OR (health N2 promotion) OR (motor N2 activit*))) NOT PT (letter OR news OR comment OR editorial OR congresses OR abstracts)

**Web of science**

TS=((((bicycle* OR "cycling " OR cyclist* OR "biking" OR "bike") NEAR/5 (lane* OR path* OR infrastructure* OR "planning" OR design* OR network* OR "space" OR "zone" OR "zones" OR track* OR dedicate*) OR Bikeabilit* OR "transport-infrastructure*" OR ("Cycle" NEAR/1 ("lane" OR "path" OR "track" OR "lanes" OR "paths" OR "tracks" OR network*)) OR (cycle NEAR/2 (infrastructure* OR dedicate* OR "path" OR "paths")) OR ((bicycle* OR "cycling " OR cyclist* OR "biking" OR "bike" ) AND ((environment* OR infrastructur* OR neighbourhood* OR neighborhood* OR roadway* OR boulevard* OR route*) NEAR/6 (interven* OR change* OR plan* OR design* OR correlat* OR factor* OR determinant* OR difference* OR structure* OR characteristic* OR measure* OR built OR role OR condition* OR context* OR improve*))))) AND (((("lifestyle" OR "life-style") NEAR/2 (modif* OR change* OR behav* OR healthy)) OR ((physical* OR transport* OR travel*) NEAR/2 (activ* OR inactiv*)) OR commut* OR leisur* OR recreat* OR ((transport* OR Prevalen* OR predictor* OR statistic* OR utilit* OR promot* OR increase* OR "play" OR "use" OR "using") NEAR/5 ("cycling" OR "bicycling" OR cyclist* OR "biking " OR "bike")) OR (behav* NEAR/2 (change* OR "healthy")) OR ("mode" NEAR/2 "travel") OR ("health" NEAR/2 "promotion") OR ("motor" NEAR/2 activit*))) ) AND DT=(article)

**Google scholar**

"bicycle|cycling |cyclist|cycle lanes|paths|infrastructure|network|space|zones|tracks" "lifestyle modification|changes|behavior"|"physical activity"|commuting|leisure|"health|healthy promotion|behavior"
