# Supplementary material for: A systematic review of the effect of infrastructural interventions to promote cycling: strengthening causal inference from observational data
Source: Int J Behav Nutr Phys Act. 2019 Oct 26;16:93. doi: 10.1186/s12966-019-0850-1 (PMC6815350; doi:10.1186/s12966-019-0850-1)
Supplement: Supplementary file 2 — Additional file 2: Appendix 2. Calculations of relative and absolute change. [file 12966_2019_850_MOESM2_ESM.docx]

**A systematic review of the effect of infrastructural interventions to promote cycling: Strengthening causal inference from observational data**

*Famke J.M. Mölenberg, Jenna Panter, Alex Burdorf, Frank J. van Lenthe*

**Appendix 2. Calculations of relative and absolute change**

**Example 1: Aittasalo(1) – Uncontrolled study**

**Outcome:** Bikes per day during afternoon peak hour

**Results - absolute change presented in text:**

Before: 646 cyclists

After: 1013 cyclists

**Results - absolute change:** 1013 – 646 = 367 cyclists/peak hour

**Results - relative change:** 1013 / 646 = 1.57 (57%)

**Example 2: Brown(2) – Controlled study**

**Outcome:** Made a bike trip on the intervention road (yes-no)

**Results - absolute change presented in text:**

Among Near residents (intervention group):

- Before: 8% were detected cycling on the intervention road
- After: 10% were detected cycling on the intervention road

Among Far residents (control group):

- Before: 5% were detected cycling on the intervention road
- After: 7% were detected cycling on the intervention road

**Results - absolute change:** (10 – 8) – (7 – 5) = 0

**Results - relative change:** (10 / 8) / (7 / 5) = 0.89 (-11%)

**References**

1. Dill J, McNeil N, Broach J, Ma L. Bicycle boulevards and changes in physical activity and active transportation: findings from a natural experiment. Prev Med. 2014;69 Suppl 1:S74-8.

2. Brown BB, Smith KR, Tharp D, Werner CM, Tribby CP, Miller HJ, et al. A Complete Street Intervention for Walking to Transit, Nontransit Walking, and Bicycling: A Quasi-Experimental Demonstration of Increased Use. J Phys Act Health. 2016;13(11):1210-9.
