# Supplementary material for: A systematic review of the effect of infrastructural interventions to promote cycling: strengthening causal inference from observational data
Source: Int J Behav Nutr Phys Act. 2019 Oct 26;16:93. doi: 10.1186/s12966-019-0850-1 (PMC6815350; doi:10.1186/s12966-019-0850-1)
Supplement: Supplementary file 3 — Additional file 3: Appendix 3. Selection of articles. [file 12966_2019_850_MOESM3_ESM.docx]

**A systematic review of the effect of infrastructural interventions to promote cycling: Strengthening causal inference from observational data**

*Famke J.M. Mölenberg, Jenna Panter, Alex Burdorf, Frank J. van Lenthe*

**Appendix 3. Selection of articles**

**Initial search (February 2018): Title and abstract screened by two researchers (n=2785)**

Screener 1: 75 papers

Screener 2: 98 papers

112 unique papers, of which 61 papers found by both screeners (54%).
After undoubling: 100 articles to be read in full-text; 28 included

**Updated search (June 2019): Title and abstract screened by one researcher (n=757)**

Screener 1: 25 papers to be read in full-text; 3 included

**The major reason for exclusion of articles retrieved in full-text (n=94):**

Code classification for exclusion:

1. no infrastructural intervention to promote cycling
2. no quantitative measure of cycling behavior (total or purpose specific)
3. no data available before and after the intervention
4. not reporting on a general adult population aged 16 and above
5. multi-component intervention, except a media campaign alongside infrastructural interventions
6. before-after, but retrospective data
7. protocol/baseline/review papers
8. no full text article available
9. data extracted from other paper with longer follow-up data available

**Table 1:** Studies excluded

| **Author (ref)** | **Main reason for exclusion** | **Code** |
| --- | --- | --- |
| Aldred (1) | No infrastructural intervention: street was closed to through motor traffic | 1 |
| Aziz (2) | No infrastructural intervention: agent-based model to predict cycling changes after a theoretical improvement in cycling infrastructure | 1 |
| Aziz (3) | No data available before and after the intervention: cross-sectional study to associate cycling infrastructure and cycling | 3 |
| Beenackers (4) | No infrastructural intervention: relocation study | 1 |
| Bhatia (5) | No quantitative measure of cycling behavior: collisions | 2 |
| Boarnet (6) | No infrastructural intervention: validation of a research method | 1 |
| Botte (7) | No infrastructural intervention: TOD (transit-oriented development), distance to public transportation | 1 |
| Brand (8) | No quantitative measure of cycling behavior: outcome of interest CO2 emissions | 2 |
| Brey (9) | No quantitative measure of cycling behavior: outcome of interest economic and social returns | 2 |
| Buehler (10) | No infrastructural intervention: review | 1 |
| Cabral (11) | No quantitative measure of cycling behavior: outcome of interest integration of the network | 2 |
| Cervero (12) | No infrastructural intervention: introduction of BART, bike rental scheme. | 1 |
| Chang (13) | No infrastructural intervention: bus rapid transit | 1 |
| Chapman (14) | Protocol of the ACTIVE study | 7 |
| Chen (15) | No quantitative measure of cycling behavior: crashes | 2 |
| Chriqui (16) | No infrastructural intervention: zoning | 1 |
| Clark (17) | No infrastructural intervention: marketing campaign promoting the use of existing trails | 1 |
| Clayton (18) | No infrastructural intervention: investigates how far infrastructural changes might enhance intrinsic motivation | 1 |
| Cope (19) | No data available before and after the intervention: cross-sectional data to show who is using the cycling facilities | 3 |
| Cope (20) | No data available before and after the intervention: cross-sectional data to show who is using the cycling facilities | 3 |
| Crane (21) | No data available before and after the intervention: cross-sectional data to show who is using the cycling facilities | 3 |
| Dill (22) | No data available before and after the intervention: cross-sectional study to associate cycling infrastructure and cycling | 3 |
| Dill (23) | No quantitative measure of cycling behavior: compliance to the rules and conflicts | 2 |
| Duncan (24) | No data available before and after the intervention: cross-sectional study observing intersections and the paths taken by cyclist | 3 |
| Flanagan (25) | No quantitative measure of cycling behavior: cycling infrastructure index as an outcome | 2 |
| Fuller (26) | No infrastructural intervention: public bicycle share program | 1 |
| Ge (27) | No infrastructural intervention: agent-based model to predict cycling changes after a theoretical improvement in cycling infrastructure | 1 |
| Giles-Corti (28) | Protocol of the RESIDential Environment Project’s study | 7 |
| Goodman (29) | Multi-component intervention: cycling lanes plus a behavioral component (cycle training) | 5 |
| Goodman (30) | No quantitative measure of cycling behavior: Who uses the infrastructure rather than changes in cycling. Iconnect study | 2 |
| Grisé (31) | No infrastructural intervention: methodology identifying where new bicycle facilities can be built | 1 |
| Gu (32) | No data available before and after the intervention: modelled the effect of additional lane miles on the expansion in bike ridership | 3 |
| Guo (33) | No infrastructural intervention: hypothetical changes in the built environment | 1 |
| Harding (34) | No quantitative measure of cycling behavior: physical activity | 2 |
| Harvey (35) | No full text article available | 8 |
| Heesch (36) | No infrastructural intervention: validation of a research method | 1 |
| Heinen (37) | No infrastructural intervention: interest was predictors of using the busway (Cambridgeshire guided busway) | 1 |
| Heinen (38) | No quantitative measure of cycling behavior: outcome of interest active transport (Cambridgeshire guided busway) | 2 |
| Heinen (39) | No infrastructural intervention: interest was baseline variability in mode choice (Cambridgeshire guided busway) | 1 |
| Heinen (40) | No quantitative measure of cycling behavior: outcome of interest modal shifts (Cambridgeshire guided busway) | 2 |
| Hipp (41) | No infrastructural intervention: validation of a research method | 1 |
| Houde (42) | No quantitative measure of cycling behavior: outcome of interest was accessibility of the cycling network | 2 |
| Jensen (43) | No quantitative measure of cycling behavior: crashes | 2 |
| Jia (44) | No infrastructural intervention: introduction of dock-less bicycle sharing | 1 |
| Jones (45) | No quantitative measure of cycling behavior: ethnograpihic study directly after opening (Cambridgeshire guided busway) | 2 |
| Jones (46) | No quantitative measure of cycling behavior: number of vehicles un specified | 2 |
| Kanani (47) | No data available before and after the intervention: only data available after the intervention | 3 |
| Karndacharuk (48) | No infrastructural intervention to promote cycling: Shared road for pedestrians | 1 |
| Kashian (49) | No quantitative measure of cycling behavior: outcome of interest was housing values | 2 |
| Kasraian (50) | No infrastructural intervention: evaluating trends in average daily distance travelled by train and its determinants | 1 |
| Keall (51) | Multi-component intervention: infrastructure investment plus behavioral components (active travel encouragement, safety education) | 5 |
| Keall (52) | No quantitative measure of cycling behavior: carbon dioxide emissions | 2 |
| Kesten (53) | No infrastructural intervention: interest was representations in the media of the busway (Cambridgeshire guided busway) | 1 |
| Kesten (54) | No infrastructural intervention: interest was experience of the new busway on active travel bahaviors (Cambridgeshire guided busway) | 1 |
| Kramer (55) | Multi-component intervention: numerous area-based initiatives but the main aim was not to promote cycling | 5 |
| Lawlor (56) | No quantitative measure of cycling behavior: description of challenges of evaluating infrastructural interventions | 2 |
| Macmillan (57) | Protocol of the Te Ara Mua-Future Streets study | 7 |
| Maques (58) | No quantitative measure of cycling behavior: outcome of interest active transport | 2 |
| McCarthy (59) | No data available before and after the intervention: cross-sectional data to show who is using the new bridge | 3 |
| Mitra (60) | Before-after, but retrospective data collection | 6 |
| Monsere (61) | Before-after, but retrospective data collection | 6 |
| Montgomery (62) | No infrastructural intervention: bus rapid transit | 1 |
| Mulvaney (63) | No quantitative measure of cycling behavior: review on injuries | 2 |
| Ngo (64) | No quantitative measure of cycling behavior: transportation energy use and greenhouse gas emissions | 2 |
| Ogilvie (65) | Protocol of the iConnect study | 7 |
| Ogilvie (66) | Review of the Cambridgeshire Guided Busway study | 7 |
| Olsen (67) | No infrastructural intervention: new motorway infrastructure | 1 |
| Panter (68) | No quantitative measure of cycling behavior: investigating environmental mechanisms (iConnect) | 2 |
| Pazin (69) | No quantitative measure of cycling behavior: leisure-time physical activity | 2 |
| Pham (70) | No infrastructural intervention: evaluating other transportation infrastructure | 1 |
| Prins (71) | No quantitative measure of cycling behavior: investigating environmental mechanisms (Cambridgeshire guided busway) | 2 |
| Rebecchi (72) | No infrastructural intervention: expected change in cycling in case of improvement and implementation of the cycling network | 1 |
| Rissel (73) | Multi-component intervention: range of community engagement and social marketing activities to promote the use of a newly completed off-road cycle paths | 5 |
| Rissel (74) | No data available before and after the intervention: survey of cycling behavior | 3 |
| Rissel (75) | Evaluates similar intervention as Crane, using similar outcomes and methodologies, but for a shorter follow-up time. | 9 |
| Roberts (76) | Protocol of the PLOT study | 7 |
| Rosas-Santizábal (77) | No infrastructural intervention: evaluating ways why cycling increased through interviews with stakeholders | 1 |
| Sadik-Khan (78) | Review paper | 7 |
| Sahlqvist (79) | No quantitative measure of cycling behavior: investigating mechanisms (iConnect) | 2 |
| Santana (80) | No infrastructural intervention of interest: pocket parks | 1 |
| Sayers (81) | Multi-component intervention: infrastructural intervention, social marketing, plus behavioral component (education) | 5 |
| Schasberger (82) | Multi-component intervention: multiple programs to promote trail use | 5 |
| Schepers (83) | No data available before and after the intervention: cross-sectional study to associate unbundling cycling traffic with bicycle usage | 3 |
| Schipperijn (84) | No infrastructural intervention of interest: bicycle playgrounds | 1 |
| Standen (85) | Before-after, but retrospective data collection | 6 |
| TenBrink (86) | Multi-component intervention: multiple programs to increase active transportation | 5 |
| Turner (87) | No full text article available | 8 |
| Van Houten (88) | No quantitative measure of cycling behavior: bicycle and motor vehicle positioning | 2 |
| Vasilev (89) | Before-after, but retrospective data collection | 6 |
| Wang (90) | No data available before and after the intervention: cross-sectional study to conduct a cost-effectiveness analysis | 3 |
| Wang (91) | No data available before and after the intervention: cross-sectional study to conduct a cost-effectiveness analysis | 3 |
| Winters (92) | Protocol of the IBIMS study | 7 |
| Yang (93) | No infrastructural intervention: review on interventions to promote cycling | 1 |
| Zaccaro (94) | No infrastructural intervention: introduction of Complete Streets programme | 1 |

**References**

1. Aldred R, Croft J. Evaluating active travel and health economic impacts of small streetscape schemes: An exploratory study in London. Journal of Transport & Health 2019;12:86-96.

2. Aziz HMA, Park BH, Morton A, Stewart RN, Hilliard M, Maness M. A high resolution agent-based model to support walk-bicycle infrastructure investment decisions: A case study with New York City. Transportation Research Part C-Emerging Technologies 2018;86:280-99.

3. Aziz HMA, Nagle NN, Morton AM, Hilliard MR, White DA, Stewart RN. Exploring the impact of walk-bike infrastructure, safety perception, and built-environment on active transportation mode choice: a random parameter model using New York City commuter data. Transportation 2018;45(5):1207-29.

4. Beenackers MA, Foster S, Kamphuis CB, et al. Taking up cycling after residential relocation: built environment factors. Am J Prev Med 2012;42(6):610-5.

5. Bhatia D, Richmond SA, Loo CKJ, Rothman L, Macarthur C, Howard A. Examining the impact of cycle lanes on cyclist-motor vehicle collisions in the city of Toronto. Journal of Transport & Health 2016;3(4):523-8.

6. Boarnet MG, Forsyth A, Day K. The street level built environment and physical activity and walking: Results of a predictive validity study for the Irvine Minnesota Inventory. Environ Behav 2011;43(6):735-75. doi: 10.1177/0013916510379760.

7. Botte M, Olaru D. Geo-spatial analysis of activity spaces in a TOD environment - Tracking impacts of rail transport policy using kernel density estimation. Road & Transport Research 2012;21(1):64-81.

8. Brand C, Goodman A, Ogilvie D. Evaluating the impacts of new walking and cycling infrastructure on carbon dioxide emissions from motorized travel: a controlled longitudinal study. Appl Energy 2014;128:284-95.

9. Brey R, Castillo-Manzano JI, Castro-Nuno M, Lopez-Valpuesta L, Marchena-Gomez M, Sanchez-Braza A. Is the widespread use of urban land for cycling promotion policies cost effective? A Cost-Benefit Analysis of the case of Seville. Land Use Policy 2017;63:130-9.

10. Buehler R, Dill J. Bikeway Networks: A Review of Effects on Cycling. Transport Reviews 2016;36(1):9-27.

11. Cabral L, Kim AM, Shirgaokar M. Low-stress bicycling connectivity: Assessment of the network build-out in Edmonton, Canada. Case Studies on Transport Policy 2019;7(2):230-8.

12. Cervero R, Caldwell B, Cuellar J. Bike-and-Ride: Build It and They Will Come. Journal of Public Transportation 2013;16(4):83-105.

13. Chang A, Miranda-Moreno L, Cao J, Welle B. The effect of BRT implementation and streetscape redesign on physical activity: A case study of Mexico City. Transportation Research Part a-Policy and Practice 2017;100:337-47.

14. Chapman R, Howden-Chapman P, Keall M, et al. Increasing active travel: aims, methods and baseline measures of a quasi-experimental study. BMC Public Health 2014;14:935. doi: 10.1186/1471-2458-14-935.

15. Chen L, Chen C, Srinivasan R. Evaluating the safety effects of bicycle lanes in New York City. … journal of public … 2012.

16. Chriqui JF, Nicholson LM, Thrun E, Leider J, Slater SJ. More Active Living-oriented County and Municipal Zoning is Associated with Increased Adult Leisure Time Physical Activity-United States, 2011. Environ Behav 2016;48(1):111-30.

17. Clark S, Bungum T, Shan GG, Meacham M, Coker L. The effect of a trail use intervention on urban trail use in Southern Nevada. Preventive Medicine 2014;67:S17-S20.

18. Clayton W, Musselwhite C. Exploring changes to cycle infrastructure to improve the experience of cycling for families. Journal of Transport Geography 2013;33:54-61.

19. Cope M, Doxford D, Hill T. Monitoring tourism on the UK's first long-distance cycle route. Journal of Sustainable Tourism 1998.

20. Cope AC, S.; Fox, K.; Lawlor, D.A.; Lockie, M.; Lumsdon, L.; Riddoch, C.; Rosen, P. The UK National Cycle Network: an assessment of the benefits of a sustainable transport infrastructure eco-logica.co.uk, 2003.

21. Crane M, Rissel C, Greaves S, Standen C, Wen LM. Neighbourhood expectations and engagement with new cycling infrastructure in Sydney, Australia: Findings from a mixed method before-and-after study. Journal of Transport & Health 2016;3(1):48-60.

22. Dill J, Carr T. Bicycle commuting and facilities in major US cities: if you build them, commuters will use them. Transportation Research Record 2003.

23. Dill J, Monsere CM, McNeil N. Evaluation of bike boxes at signalized intersections. Accid Anal Prev 2012;44(1):126-34.

24. Duncan A. A comparative analysis of cyclists' paths through shared space and non-shared intersections in Coventry, England. Journal of Urban Design 2017;22(6):833-44.

25. Flanagan E, Lachapelle U, El-Geneidy A. Riding tandem: Does cycling infrastructure investment mirror gentrification and privilege in Portland, OR and Chicago, IL? Research in Transportation Economics 2016;60:14-24.

26. Fuller D. Potential of built environment interventions involving deployment of public bicycles to increase utilitarian cycling: The case of BIXI© in Montreal, Quebec. Dissertation Abstracts International: Section B: The Sciences and Engineering 2013;74(4-B(E):No Pagination Specified.

27. Ge JQ, Polhill JG. Exploring the Combined Effect of Factors Influencing Commuting Patterns and CO2 Emissions in Aberdeen Using an Agent-Based Model. Jasss-the Journal of Artificial Societies and Social Simulation 2016;19(3).

28. Giles-Corti B, Knuiman M, Pikora TJ, et al. Can the impact on health of a government policy designed to create more liveable neighbourhoods be evaluated? An overview of the RESIDential Environment Project. N S W Public Health Bull 2007;18(11-12):238-42.

29. Goodman A, Panter J, Sharp SJ, Ogilvie D. Effectiveness and equity impacts of town-wide cycling initiatives in England: A longitudinal, controlled natural experimental study. Soc Sci Med 2013;97:228-37. doi: 10.1016/j.socscimed.2013.08.030.

30. Goodman A, Sahlqvist S, Ogilvie D. Who uses new walking and cycling infrastructure and how? Longitudinal results from the UK iConnect study. Prev Med 2013;57(5):518-24. doi: 10.1016/j.ypmed.2013.07.007.

31. Grise E, El-Geneidy A. If we build it, who will benefit? A multi-criteria approach for the prioritization of new bicycle lanes in Quebec City, Canada. Journal of Transport and Land Use 2018;11(1):217-35.

32. Gu J, Mohit B, Muennig PA. The cost-effectiveness of bike lanes in New York City. Inj Prev 2017;23(4):239-43.

33. Guo JY, Gandavarapu S. An economic evaluation of health-promotive built environment changes. Prev Med 2010;50 Suppl 1:S44-9.

34. Harding MC, Bott QD, Jonas CE. The Mālaekahana Path: An Ecological Model-Based Intervention for Increasing Walking and Biking in Rural Hawai'i. J Phys Act Health 2017;14(12):965-7. doi: 10.1123/jpah.2017-0330.

35. Harvey E, Brown CT, DiPetrillo S, Kay A. Bicycling to Rail Stations in New Jersey. Transportation Research Record 2016(2587):50-60.

36. Heesch KC, Langdon M. The usefulness of GPS bicycle tracking data for evaluating the impact of infrastructure change on cycling behaviour. 2016.

37. Heinen E, Panter J, Dalton A, Jones A, Ogilvie D. Sociospatial patterning of the use of new transport infrastructure: Walking, cycling and bus travel on the Cambridgeshire guided busway. J Transp Health 2015;2(2):199-211.

38. Heinen E, Panter J, Mackett R, Ogilvie D. Changes in mode of travel to work: a natural experimental study of new transport infrastructure. International Journal of Behavioral Nutrition & Physical Activity 2015;12:81. doi: 10.1186/s12966-015-0239-8.

39. Heinen E, Ogilvie D. Variability in baseline travel behaviour as a predictor of changes in commuting by active travel, car and public transport: a natural experimental study. J Transp Health 2016;3(1):77-85.

40. Heinen E, Harshfield A, Panter J, Mackett R, Ogilvie D. Does exposure to new transport infrastructure result in modal shifts? Patterns of change in commute mode choices in a four-year quasi-experimental cohort study. J Transp Health 2017;6:396-410.

41. Hipp J, Adlakha D, Eyler AA, Chang B, Pless R. Emerging technologies: Webcams and crowd-sourcing to identify active transportation. American Journal of Preventive Medicine 2013;44(1):96-7.

42. Houde M, Apparicio P, Seguin AM. A ride for whom: Has cycling network expansion reduced inequities in accessibility in Montreal, Canada? Journal of Transport Geography 2018;68:9-21.

43. Jensen SU. Safety Effects of Converting Intersections to Roundabouts. Transportation Research Record 2013(2389):22-9.

44. Jia Y, Ding D, Gebel K, et al. Effects of new dock-less bicycle-sharing programs on cycling: A retrospective study in Shanghai. BMJ Open 2019;9(2). doi: 10.1136/bmjopen-2018-024280.

45. Jones CH, Cohn S, Ogilvie D. Making sense of a new transport system: an ethnographic study of the Cambridgeshire Guided Busway. PLoS ONE 2013;8(7):e69254.

46. Jones DL. The behavioral impacts of urban street modifications: A case study of East Blvd. in Charlotte, NC. Dissertation Abstracts International Section A: Humanities and Social Sciences 2014;74(7-A(E):No Pagination Specified.

47. Kanani A, North R. Delivering the Exe Estuary Trail cycling and walking route, UK. Proceedings of the Institution of Civil Engineers-Municipal Engineer 2017;170(2):116-24.

48. Karndacharuk A, Wilson DJ, Dunn RCM. Analysis of Pedestrian Performance in Shared-Space Environments. Transportation Research Record 2013(2393):1-11.

49. Kashian R, Winden M, Storts E. The Effects of a Recreational Bike Path on Housing Values in Muskego, Wisconsin. Journal of Park and Recreation Administration 2018;36(3):160-73.

50. Kasraian D, Maat K, van Wee B. Urban developments and daily travel distances: Fixed, random and hybrid effects models using a Dutch pseudo-panel over three decades. Journal of Transport Geography 2018;72:228-36.

51. Keall M, Chapman R, Howden-Chapman P, Witten K, Abrahamse W, Woodward A. Increasing active travel: results of a quasi-experimental study of an intervention to encourage walking and cycling. J Epidemiol Community Health 2015;69(12):1184-90. doi: 10.1136/jech-2015-205466.

52. Keall MD, Shaw C, Chapman R, Howden-Chapman P. Reductions in carbon dioxide emissions from an intervention to promote cycling and walking: A case study from New Zealand. Transportation Research Part D-Transport and Environment 2018;65:687-96.

53. Kesten JM, Cohn S, Ogilvie D. The contribution of media analysis to the evaluation of environmental interventions: the commuting and health in Cambridge study. BMC Public Health 2014;14:482. doi: 10.1186/1471-2458-14-482.

54. Kesten JM, Guell C, Cohn S, Ogilvie D. From the concrete to the intangible: understanding the diverse experiences and impacts of new transport infrastructure. International Journal of Behavioral Nutrition & Physical Activity 2015;12:72. doi: 10.1186/s12966-015-0230-4.

55. Kramer D, Droomers M, Jongeneel-Grimen B, Wingen M, Stronks K, Kunst AE. The impact of area-based initiatives on physical activity trends in deprived areas; a quasi-experimental evaluation of the Dutch District Approach. International Journal of Behavioral Nutrition & Physical Activity 2014;11(1). doi: 10.1186/1479-5868-11-36.

56. Lawlor DA, Ness AR, Cope AM, Davis A, Insall P, Riddoch C. The challenges of evaluating environmental interventions to increase population levels of physical activity: The case of the UK National Cycle Network. J Epidemiol Community Health 2003;57(2):96-101. doi: 10.1136/jech.57.2.96.

57. Macmillan AK, Mackie H, Hosking JE, et al. Controlled before-after intervention study of suburb-wide street changes to increase walking and cycling: Te Ara Mua-Future Streets study design. BMC Public Health 2018;18(1):850. doi: 10.1186/s12889-018-5758-1.

58. Marques R, Hernandez-Herrador V, Calvo-Salazar M, Garcia-Cebrian JA. How infrastructure can promote cycling in cities: Lessons from Seville. Research in Transportation Economics 2015;53:31-44.

59. McCarthy D. Perceptions about and use of a new scenic bridge path among walkers, runners, and cyclists in lowcountry South Carolina. Preventive Medicine: An International Journal Devoted to Practice and Theory 2010;51(1):94-5.

60. Mitra R, Ziemba RA, Hess PM. Mode substitution effect of urban cycle tracks: Case study of a downtown street in Toronto, Canada. International Journal of Sustainable Transportation 2017;11(4):248-56.

61. Monsere CM, McNeil N, Dill J. Multiuser Perspectives on Separated, On-Street Bicycle Infrastructure. Transportation Research Record 2012(2314):22-30.

62. Montgomery BN. Cycling Trends and Fate in the Face of Bus Rapid Transit Case Study of Jinan, Shandong Province, China. Transportation Research Record 2010(2193):28-36.

63. Mulvaney CA, Smith S, Watson MC, et al. Cycling infrastructure for reducing cycling injuries in cyclists. Cochrane Database Syst Rev 2015;12:CD010415. doi: 10.1002/14651858.CD010415.pub2.

64. Ngo VD, Frank LD, Bigazzi AY. Effects of new urban greenways on transportation energy use and greenhouse gas emissions: A longitudinal study from Vancouver, Canada. Transportation Research Part D-Transport and Environment 2018;62:715-25.

65. Ogilvie D, Bull F, Cooper A, et al. Evaluating the travel, physical activity and carbon impacts of a 'natural experiment' in the provision of new walking and cycling infrastructure: Methods for the core module of the iConnect study. BMJ Open 2012;2(1). doi: 10.1136/bmjopen-2011-000694.

66. Ogilvie D, Panter J, Guell C, Jones A, Mackett R, Griffin S. Health impacts of the Cambridgeshire Guided Busway: a natural experimental study. 2016.

67. Olsen JR, Mitchell R, Ogilvie D, team Ms. Effects of new motorway infrastructure on active travel in the local population: a retrospective repeat cross-sectional study in Glasgow, Scotland. Int j behav nutr phys act 2016;13:77.

68. Panter J, Ogilvie D, iConnect c. Theorising and testing environmental pathways to behaviour change: natural experimental study of the perception and use of new infrastructure to promote walking and cycling in local communities. BMJ Open 2015;5(9):e007593.

69. Pazin J, Garcia LMT, Florindo AA, et al. Effects of a new walking and cycling route on leisure-time physical activity of Brazilian adults: A longitudinal quasi-experiment. Health & Place 2016;39:18-25.

70. Pham T, Riley E, Harris P. Inclusion of Health in Environmental Impact Assessment of Major Transport Infrastructure Projects in Vietnam. Int J Health Policy Manag 2018;7(9):828-35. doi: 10.15171/ijhpm.2018.36.

71. Prins RG, Panter J, Heinen E, Griffin SJ, Ogilvie DB. Causal pathways linking environmental change with health behaviour change: Natural experimental study of new transport infrastructure and cycling to work. Prev Med 2016;87:175-82. doi: 10.1016/j.ypmed.2016.02.042.

72. Rebecchi A, Boati L, Oppio A, Buffoli M, Capolongo S. Measuring the expected increase in cycling in the city of Milan and evaluating the positive effects on the population's health status: a Community-Based Urban Planning experience. Ann Ig 2016;28(6):381-91.

73. Rissel CE, New C, Wen LM, Merom D, Bauman AE, Garrard J. The effectiveness of community-based cycling promotion: Findings from the cycling connecting communities project in Sydney, Australia. International Journal of Behavioral Nutrition & Physical Activity 2010;7. doi: 10.1186/1479-5868-7-8.

74. Rissel C, Merom D, Bauman A, Garrard J, Wen LM, New C. Current cycling, bicycle path use, and willingness to cycle more-findings from a community survey of cycling in Southwest Sydney, Australia. J Phys Act Health 2010;7(2):267-72.

75. Rissel C, Greaves S, Li Ming W, Crane M, Standen C. Use of and short-term impacts of new cycling infrastructure in inner-Sydney, Australia: a quasi-experimental design. International Journal of Behavioral Nutrition & Physical Activity 2015;12:1-8. doi: 10.1186/s12966-015-0294-1.

76. Roberts JD, Hu M, Saksvig BI, Brachman ML, Durand CP. Examining the influence of a new light rail line on the health of a demographically diverse and understudied population within the washington, D.C. metropolitan area: A protocol for a natural experiment study. Int J Environ Res Public Health 2018;15(2). doi: 10.3390/ijerph15020333.

77. Rosas-Satizabal D, Rodriguez-Valencia A. Factors and policies explaining the emergence of the bicycle commuter in Bogota. Case Studies on Transport Policy 2019;7(1):138-49.

78. Sadik-Khan J, Solomonow S. Improving Public Health by Making Cities Friendly to Walking and Biking: Safer, More Active Transportation Starts With the Street. JAMA Intern Med 2017;177(5):613-4.

79. Sahlqvist S, Goodman A, Jones T, Powell J, Yena S, Ogilvie D. Mechanisms underpinning use of new walking and cycling infrastructure in different contexts: mixed-method analysis. International Journal of Behavioral Nutrition & Physical Activity 2015;12:1-15. doi: 10.1186/s12966-015-0185-5.

80. Santana DT, Rechia S, Rodrigues E. The Cracks of the City: The "Praca De Bolso Do Ciclista" in Curitiba, Brazil. Movimento 2017;23(1):311-24.

81. Sayers SP, Lemaster JW, Thomas IM, Petroski GF, Ge B. Bike, walk, and wheel: A way of life in Columbia, Missouri, revisited. Am J Prev Med 2012;43(5 SUPPL.4):S379-S83. doi: 10.1016/j.amepre.2012.07.006.

82. Schasberger MG, Hussa CS, Polgar MF, McMonagle JA, Burke SJ, Gegaris Jr AJ. Promoting and developing a trail network across suburban, rural, and urban communities. Am J Prev Med 2009;37(6 Suppl 2):S336-44.

83. Schepers P, Heinen E, Methorst R, Wegman F. Road safety and bicycle usage impacts of unbundling vehicular and cycle traffic in Dutch urban networks. European Journal of Transport and Infrastructure Research 2013;13(3):221-38.

84. Schipperijn J, Hansen CK, Rask S. Use and activity levels on newly built bicycle playgrounds. Urban Forestry & Urban Greening 2015;14(1):163-9.

85. Standen C, Crane M, Collins A, Greaves S, Rissel C. Determinants of mode and route change following the opening of a new cycleway in Sydney, Australia. Journal of Transport & Health 2017;4:255-66.

86. TenBrink DS, McMunn R, Panken S. Project U-Turn: increasing active transportation in Jackson, Michigan. Am J Prev Med 2009;37(6 Suppl 2):S329-35.

87. Turner D. Red Routes in London - Not just red lines. Proceedings of the Institution of Civil Engineers-Transport 1997;123(3):151-62.

88. Van Houten R, Seiderman C. How pavement markings influence bicycle and motor vehicle positioning - Case study in Cambridge, Massachusetts. Edtion ed. Bicycles and Pedestrians; Developing Countries 2005. Washington: Transportation Research Board Natl Research Council, 2005:3-14.

89. Vasilev M, Pritchard R, Jonsson T. Trialing a Road Lane to Bicycle Path RedesignChanges in Travel Behavior with a Focus on Users' Route and Mode Choice. Sustainability 2018;10(12).

90. Wang G, Macera CA, Scudder-Soucie B, Schmid T, Pratt M, Buchner D. Cost effectiveness of a bicycle/pedestrian trail development in health promotion. Prev Med 2004;38(2):237-42. doi: 10.1016/j.ypmed.2003.10.002.

91. Wang G, Macera CA, Scudder-Soucie B, et al. Cost analysis of the built environment: the case of bike and pedestrian trials in Lincoln, Neb. Am J Public Health 2004;94(4):549-53.

92. Winters M, Branion-Calles M, Therrien S, et al. Impacts of Bicycle Infrastructure in Mid-Sized Cities (IBIMS): protocol for a natural experiment study in three Canadian cities. BMJ Open 2018;8(1):e019130.

93. Yang L, Sahlqvist S, McMinn A, Griffin SJ, Ogilvie D. Interventions to promote cycling: systematic review Review. BMJ 2010;341:c5293.

94. Zaccaro HN, Atherton E. Bright spots, physical activity investments that work-Complete Streets: redesigning the built environment to promote health. Br J Sports Med 2018;52(18):1168-9. doi: 10.1136/bjsports-2017-097717.
