# Supplementary material for: A systematic review of the effect of infrastructural interventions to promote cycling: strengthening causal inference from observational data
Source: Int J Behav Nutr Phys Act. 2019 Oct 26;16:93. doi: 10.1186/s12966-019-0850-1 (PMC6815350; doi:10.1186/s12966-019-0850-1)
Supplement: Supplementary file 4 — Additional file 4: Table S1. Summary of the results. [file 12966_2019_850_MOESM4_ESM.docx]

**A systematic review of the effect of infrastructural interventions to promote cycling: Strengthening causal inference from observational data**

*Famke J.M. Mölenberg, Jenna Panter, Alex Burdorf, Frank J. van Lenthe*

**Supplemental table 1**: Summary of the results

|  | Cycling behavior | | |  | Usage of infrastructure | | |
| --- | --- | --- | --- | --- | --- | --- | --- |
|  | Outcomes reported | In favor of the intervention | Median relative change^a^ |  | Outcomes reported | In favor of the intervention | Median relative change^a^ |
|  | n | n | % |  | n | n | % |
| All outcomes | 52 | 38 | NA |  | 21 | 21 | NA |
| All outcomes, quantitative summary | 36 | 29 | 23 |  | 20 | 20 | 62 |
|  |  |  |  |  |  |  |  |
| Study design |  |  |  |  |  |  |  |
| Controlled | 18 | 13 | 19 |  | 4 | 4 | 143 |
| Uncontrolled | 18 | 16 | 30 |  | 16 | 16 | 60 |
|  |  |  |  |  |  |  |  |
| Exposure time |  |  |  |  |  |  |  |
| ≥1 year | 17 | 16 | 40 |  | 12 | 12 | 55 |
| <1 year | 19 | 13 | 15 |  | 8 | 8 | 93 |
|  |  |  |  |  |  |  |  |
| Data collection methods |  |  |  |  |  |  |  |
| Objective measures^c^ | 7 | 3 | -1 |  | 8^e^ | 8 | 39 |
| Subjective measures^d^ | 29 | 26 | 27 |  | 8 | 8 | 73 |
|  |  |  |  |  |  |  |  |
| Statistical analyses |  |  |  |  |  |  |  |
| Tested | 26 | 22 | 12 |  | 6 | 6 | 163 |
| Not tested | 10 | 7 | 7 |  | 14 | 14 | 60 |

^a^ No units were presented for the median relative change because it can refer to various metrics.

^c^ Objective registration methods included GPS and accelerometer data for studies assessing cycling behavior, and

GPS, accelerometer data and automatic counting stations for studies assessing usage of the infrastructure.

^d^ Subjective registration methods included self-reported cycling by travel diaries, telephone interviews, and surveys for studies assessing cycling behavior, and direct observations for studies assessing usage of the infrastructure.

^e^ For four outcomes it was unclear what type of data collection method was used, or methods changed over time.
